# Programmed Cell Death via Type IV Photodynamic Therapy Using Internalized Two-Photon Activated Molecular Nanomachines

**DOI:** 10.1021/acsabm.5c01318

**Published:** 2025-10-13

**Authors:** Thomas. S. Bradford, Dongdong Liu, James M. Tour, Robert Pal

**Affiliations:** † Department of Chemistry, 3057 Durham University, South Road, Durham DH1 3LE, United Kingdom; ‡ Department of Chemistry, 3990 Rice University, Houston, Texas 77005, United States; § Department of Chemistry Department of Materials Science and NanoEngineering Rice Advanced Materials Institute and Smalley-Curl Institute NanoCarbon Center, Rice University, Houston, Texas 77005, United States

**Keywords:** nanomachines, MNM, PDT, multiphoton, 2PE, NIR activation

## Abstract

Direct photodynamic therapy (PDT) is a growing research area currently being explored as an alternative treatment for various cancers. Compared to traditional, indirect PDT, which exploits the reaction of oxygen with the photosensitizer (PS) to damage specially targeted cells, direct PDT utilizes the PS itself to disrupt the target cell, meaning no reactive oxygen species (ROS) are generated. The activation of Type IV technologies specifically induces a structural change within the photosensitizer, resulting in the activation of its therapeutic effect. In contrast to traditional invasive surgeries, chemotherapy, or ROS-based methods, direct methods of PDT pose significantly less damaging off-target effects. Here, we propose an exciting extension of our prior reported, near-infrared light-activated, molecular nanomachines (MNMs), previously shown to promote cell-specific necrosis via disruption of cellular membranes. We show that the modification of MNMs with polyethylene glycol (PEG), or triphenol phosphonium (TPP+) containing functional groups, allows for homeostatic crossing of the phospholipid bilayer and localization at the mitochondrial membrane. By subsequent activation of the rotor from within the targeted cells, we present the ability to eliminate cells without triggering necrotic cell death, instead inducing an additional mechanism of programmed cell death (PCD), while maintaining the integrity of the cellular membrane, thus enacting a significantly cleaner, more therapeutically favorable mode of inducing cell death. A significant development is in the use of light-activated molecular machines for cancer treatments, with a single MNM-based technology being able to access both necrotic and non-necrotic modes of cell elimination by simply switching the excitation procedure.

## Graphical abstract

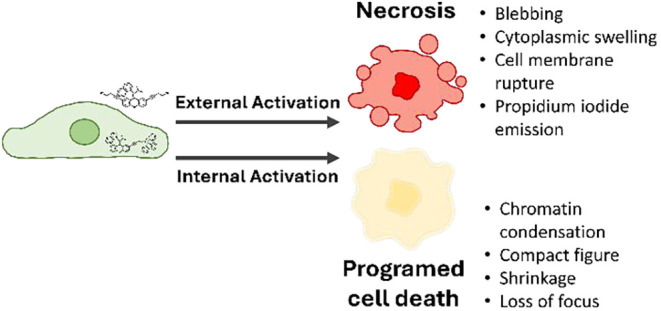

## Introduction

The most recently compiled data from the World Health Organization’s International Agency for Research on Cancer, collated in 2022, states that the global incidence of all cancers totals 19,976,499—with a mortality of 9,743,832 *per annum*.^1^ Due to this prevalence, there is great multidisciplinary interest in the development of additional modes of personalized targeted mechanics of efficient cancerous cell elimination. The overarching goal of the scientific community is the circumvention of off-target damages commonly found with more traditional chemotherapies, including both neuro- and renal toxicities.^2^ This also includes the suppression of white blood cell production leading to reduced immunity and heightened susceptibility to secondary disease.^3^ Photodynamic therapy (**PDT**) has been an extensively studied field for the treatment of various types of cancers since the 1970s,^4^ but the more recent development of type IV mechanisms—those in which a structural change within the photosensitizer is triggered upon light excitation—pose a promising avenue for the next generation cancer therapeutics.^5^

Using modified, second-generation, light-activated Feringa motors^6^—known as molecular nanomachines (**MNMs**)—we previously reported that the 355–365 nm ultraviolet (**UV**) induced *cis–trans* isomerization within the rotor (2–3 MHz) can be utilized to penetrate cell membranes (MNM 1, Figure 1).^7^ This was further extended to disrupt bacterial cell walls, destroy tissue, and kill multicellular eukaryotes within subsequent works, illustrating the potential of the technology.^8,9^ Further synthetic modifications have also been previously presented, allowing for near-UV 405 nm wavelength-induced rotation to trigger the necrotic cell death of pancreatic cancer cells.^10^ However, due to the relatively high energy activation wavelengths employed, any proposed UV or near-UV activated PDT agent carries the innate limitation of causing secondary cellular damage. This significantly reduces the range of possible future treatments that can be accessed by the technology. In an effort to address this handicap, we recently devised a more biologically preferred, and less destructive, low-energy near-infrared (**NIR**) activation method through two-photon excitation (**2PE**) at 710–720 nm in a three-dimensional (**3D**) raster pattern.^11^

**Figure 1:**
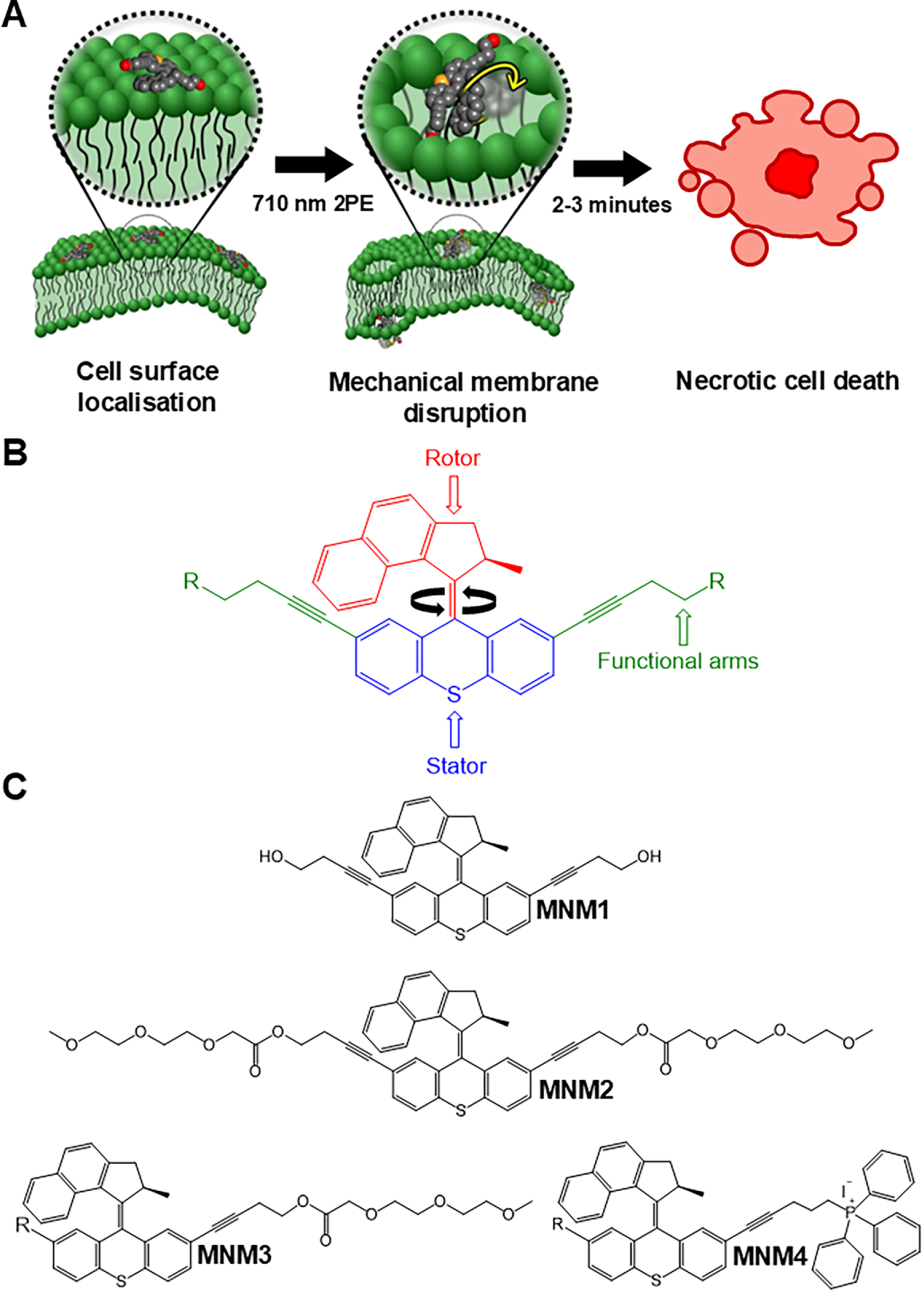
MNM structures for 2PE-induced (710 nm) apoptosis via mechanical action upon organelles. (A) Schematic of previously determined NIR 2PE-induced crossing of cell surface localized MNMs via nanomechanical disruption of the cellular membrane. Adapted from Garcia-Lopex et al.^7^ (B) A representative schematic showing the general structure of a MNM based upon a second-generation Feringa motor with the rotor highlighted in red, stator in blue, and functionalization sites in green. Upon activation, the *cis–trans* isomerization about the central axis causes the rotor to spin unidirectional about the stator at a previously measured 2–3 MHz. (C) Detailed chemical structure of the MNNMs utilized within this work. MNM 1 is the fast (2–3 MHz) noninternalizing motor used in previous works, MNMs 2 and 3 functionalized with PEG moieties on two and one functional arms, respectively, whereas MNM 4 is functionalized with TPP+ on a single arm (for MNMs 3 and 4 R = H leaving one arm available for target vector (-R) functionalization). The syntheses are described in the Supporting Information, alongside NMR characterization data, and are all found within our previous publications.

Cell death has long been categorized into two overarching groups, with the first references to distinct cell death pathways being found as early as 1951 when Glucksmann coined the term “programmed cell death” (**PCD**) to refer to cell death that occurred at specifically defined stages of the normal cell development cycle.^12^ In the 1970s, this idea was extended when histochemical studies on lysosomal changes provided the first evidence for two distinct modalities for the mortality of mammalian cells, initially interpreted as two forms of necrotic death, regular and shrinkage necrosis.^13^ The term apoptosis was first introduced in 1972,^14^ and now necrosis and apoptosis are fully understood to be two separate and distinct cell death pathways. Crucially, for the development of the next generation cancer therapeutics, apoptosis and other mechanisms of PCD have the benefit of producing little to no immune response from the body, utilizing the shuttling of phosphatidylserine to the cell surface to signal macrophage clean up. These types of cell death are therefore reported to trigger little to no inflammation.^15^ Necrotic, or unscheduled, cell death, on the other hand, leads to a spillage of intracellular proteins into the extracellular space, triggering a damage response from the host’s immune system. While some cancers and cancer treatments may benefit from an induced immune response from necrotic cell death, if coadministered with forms of immunotherapy,^16^ most treatments aim for an induction of cleaner PCD pathways to reduce the onset of harmful side effects.^17^

Our previous work has focused on anchoring MNMs to the cell surface, before excitation and mechanical disruption of the cell membrane, followed by subsequent irreversible necrotic cell death.^7^ While capable of targeted, cell-type-specific elimination, the medical application of treatments resulting in necrosis of the targeted area remains limited. Many previously reported drug-like molecules exhibit remarkable dose–response relationships for induced modes of cell death. The redox cycling agent 2,3-dimethoxy-1,4-napthoquinone (**DMNQ**) is a common example, inducing cell proliferation, apoptosis, or necrosis depending on the administered concentration.^18^ While this mechanism cannot be directly compared to MNM-induced cell death, as even low μM concentrations of MNMs, when activated on the cell surface, disrupt the target membrane and lead to necrotic cell death, it does provide inspiration.

In addition, the differing mechanisms of induced cell death exhibited by direct PDTs (both type I and II) also show a basis within the current literature for using light-activated MNMs to induce multiple routes of cell death depending on the excitation protocol used. In type I and II direct PDT, the accumulation of photosensitizer (**PS**) molecules within cell’s plasma membrane has been shown to lead to necrosis.^19^ Conversely, if the PS is shown to localize about the cell’s mitochondria, or other organelles, various programmed death pathways such as apoptosis have been observed.^20^ Based on this, we propose that if MNMs can be redesigned to efficiently cross the phospholipid bilayer via natural homeostatic uptake, subsequently anchored to intracellular target receptors or organelles, and be momentarily activated from within, sufficient internal damage may be caused without significantly damaging the cell’s membrane. This could trigger the much favored and clinically accepted apoptotic or other PCD pathways as a mode of MNM-induced action of cell death.

Herein, investigations into biologically safe, NIR activated MNNs continue with a new family of compounds designed to purposefully internalize within the cells studied, with the aim of mechanically inducing cell death solely via controlled internal cellular damage without rupture to the outer cellular membrane, resulting in targeted cell elimination by apoptosis as opposed to the previously developed necrotic pathway in our previous work. Two variants of commonly utilized functionalizations known to promote cellular uptake of drug-like compounds are incorporated into the core MNMs design: poly­(ethylene glycol) (**PEG**) and triphenyl-phosphonium (**TTP+**) functionalization. PEGylation has long been used within drug delivery to alter pharmacokinetic and pharmacodynamic properties and promote efficient bilayer permeability.^21^ MNMs 2 and 3 (Figure 1) are PEGylated on two and one functional arm, respectively, to both investigate the ability of PEG addition to promote internalization and to determine if one side of the compound can be left free for further functionalizations, such as specific protein or intracellular organelle targeting, while maintaining nondisruptive live-cell penetration. TPP+ functionalization is commonly used as a mitochondriotropic carrier, not only to promote crossing of the cellular membrane but also to localize functionalized drug-like compounds at the negatively charged mitochondrial network.^22^ The promising ability of various reported TPP+ conjugates to cross the phospholipid bilayer and localize within the mitochondria, in addition to improving generic pharmacokinetic properties, makes this functionalization an exciting route to explore for MNM modification.^23^ As such, MNM 4 (Figure 1) was developed and is investigated, alongside the PEGylated machines, for its internalization capability and ability to promote PCD via internal activation of the rotor while bound to the mitochondrial network.

## Experimental Section

### Cell Culture

NIH 3T3 mouse embryonic fibroblasts (CRL-1658) were grown as a single monolayer in GibcoTM Dulbecco’s modified Eagle’s medium (**DMEM**) with GlutaMAXTM/F12 supplemented with 10% fetal bovine serum (**FBS**) in 75 cm^2^ plastic culture flasks, with no prior surface treatment. Incubation was carried out at 37 °C and 5% (v/v) CO_2_, and under average humidity conditions. Cell harvesting was carried out via washing with 10% phosphate-buffered saline (**PBS**) prior to the addition of trypsin solution (0.25%). 5–10 min incubation at 37.5 °C was carried out prior to resuspension in fresh media by repeated aspiration with a sterile plastic pipet.

Cells were defrosted from stocks, kept frozen at −78 °C in freezing media GibcoTM Dulbecco’s modified Eagle’s medium with GlutaMAXTM/F12 supplemented with 10% fetal bovine serum with the addition of 10% DMSO, by immediate warming with gloved hand until slightly defrosted to a level where the frozen cellular pellet could move freely in the cryovial, followed by immediate addition to a prewarmed to 37 °C 75 cm^2^ plastic culture flask. Before incubation overnight, cells adhere fully.

### Microscope Slide Preparation

Microscopy cells were seeded by the addition of 100 μL of concentrated cellular suspension in growth media, acquired by trypsinization of the stock solutions, onto untreated surface iBibi 500 μL live-cell channel slides, followed by the addition of 400 μL of fresh growth media into each well and allowed to grow to 50% confluence, at 37 °C in 5% CO_2_, 10% humidity. Following cell adherence and division on the slide surface (an average of 48 h), the growth media was replaced, and cells were treated with the studied nanomachines (0.5–1 μM) and propidium iodide/Annexin V Alexa Fluor 488 conjugate stain (100 nM), ensuring DMSO concentration present in the final imaging medium never crossed 0.1% v/v. For live-cell imaging, DMEM GlutaMAX/F12 media (10% FBS) lacking phenol red was used from this point onward to prevent unwanted UV-induced fluorescence from the pH indicator. Following incubation, the channels were washed with live-cell imaging media and imaged using a purposely built incubator housing the microscope maintaining 37 °C, 5% CO_2_, and 10% humidity.

Two methods were used for MNM incubation and staining procedures for investigating TTN for solutions with MNM in solution, or internalized within the sample: standard method—the studied MNM were loaded into the channel slides containing 500 μL cell media, by addition of 2.5 μL (for 0.5 μM final concentration–single-photon studies) or 5 μL (for 1 μM final concentration—two-photon studies) of 1 mM stock solution and left to incubate at 37 °C in 5% CO_2_ for 30 min, followed by staining with the required fluorescent dyes, 100 nM PI or 100 nM AV, followed by immediate transfer to the microscope incubator housing and subsequent image acquisition. Internalization method—Studied MNMs were loaded channel slides containing 500 μL cell media, by addition of 2.5 μL (for 0.5 μM final concentration, single-photon studies) or 5 μL (for 1 μM final concentration, two-photon studies) of 1 mM stock solution and left to incubate for various stated time frames ranging from 30 min to 24 h. Followed by 3× washes with 1 mL of fresh MNM-free DMEM, GlutaMAX/F12 media (10% FBS) lacking phenol red was added to one well of the channel slides, tilted, and left to run through the channel, and removed from the opposite well. Before subsequent staining with the required fluorescent dyes, 100 nM PI or 100 nM AVAF, the reaction was followed by immediate transfer to the microscope incubator housing and image acquisition. Each MNM stock solution was in DMSO with final DMSO concentrations not exceeding 0.1% in the final imaging media solution to avoid unwanted cell membrane permeabilization. It should be noted that all MNM loading experiments were carried out in a light-suppressed environment, and the possibility of induced or accelerated uptake due to interaction between the molecular motors and the applied costain has been eliminated previously using a series of individual and reversed loading experiments.

### Laser Scanning Confocal Microscopy Procedures

All live-cell microscopy experiments were performed on a custom-built PhMoNa system^24^ based on a Leica SP5 II (DMI6000 inverted chassis) LSCM platform containing integrated visible laser lines: 458, 476, 488, 496, 514 nm argon and 543, 633 nm HeNe. In addition to a fiber-coupled 355 nm coherent laser (Coherent Genesis, Nd:YAG third harmonic, 80 mW) for UV activation of the MNMs, and a femtosecond pulsed coherent chameleon vision ii Ti:sapphire tunable (Coherent Chameleon Vision 680–1080 nm, 65 mW @ 710 nm, 80 MHz, 100 fs) laser, with additional EOM power control, for NIR rotor activation. The modular PhMoNa technique is based on a laser scanning confocal microscope (**LSCM**) harnessing spatially modulated illumination intensities, using an in situ-generated raster-scanned standing wave excitation beam optical grid pattern. The threshold algorithm to control brightness automated by the Leica LAX software is calculated by the image-specific signal-to-noise ratio.

### Single-Photon 355 nm UV MNM Activation Imaging Procedure

Steady-state fluorescence images were recorded using the PhMoNa enhanced Leica SP5 II LSCM confocal microscope equipped with an HCX PL APO 63×/1.40 NA LambdaBlue Oil immersion objective. Data were collected using 2× digital magnification at 100 Hz/line scan speed (4-line average, bidirectional scanning) at 355 nm (Coherent Genesis, third harmonic NdYAG laser, set at 20 mW, 400 nJ/voxel total dwell time). In order to achieve excitation with maximal probe emission, the microscope was equipped with a triple-channel imaging detector comprising a conventional PMT system and two HyD hybrid avalanche photodiode detectors. The frame size was determined at 1024 × 1024 pixel, with ×2 digital magnification to ensure illumination flatness of field and 0.6 airy disc unit (AU) determining the applied pinhole diameter rendering on voxel to correspond to 62 × 62 nm^2^ (frame size 125 × 125 μm^2^) with a section thickness set at 188 nm (at 355 nm excitation). HeNe and argon integrated ion lasers were used to aid parallel transmission image capture of the PI and other loaded stain signals, used to follow the onset of necrosis and other cell death pathways. All imaging parameters are kept constant across experiments; this includes voxel size, laser power, line speed, and averaging sequences unless otherwise noted within this work.

### Two-Photon 710 nm NIR MNM Activation Imaging Procedure

Live-cell Multiphoton microscopy experiments were performed on the same custom-built PhMoNa system based on a Leica SP5 II (DMI6000 inverted chassis) LSCM platform operating with a Coherent Chameleon Vision II tunable (680–1080 nm, 65 mW @ 710 nm, 80 MHz, 100 fs) multiphoton laser for NIR activation of the motor, using an ×20 0.95 NA oil immersion objective operating at unidirectional 100 Hz scan speed with 2-line accumulation unidirectional 1024 × 1024 pixel FOV for sequential MP nanomachine activation and PI imaging. 2PE activation of the MNMs was achieved using an optimized ×3.5 digital zoom to provide a 120 × 120 μm FOV in order to study 1–3 cells simultaneously and provide sufficient continuous NIR laser scanning with sufficiently long integration time for each pixel, so an adequate photon flux can be delivered to promote nonlinear MNM activation. Scan speeds for NIR activation were set at a unidirectional rate of 100 Hz with the above detailed scan settings. A five-step z-stack, with a 1 μm step size, was enacted to ensure consistent results regardless of cell morphology and compensate for chromatic aberration between the integrated 543 nm HeNe ion and Coherent Chameleon Vision II tunable (680–1080 nm, 65 mW @ 710 nm, 80 MHz, 100 fs) multiphoton lasers.

### Post-Processing

All experiments and results have been measured with a minimum of 5 distinct field of views/cell populations evaluated. Representative images are shown for each experiment, with results consistently reproduced within 3% standard deviation. All post-image processing was carried out on the open-source, plugin prepacked, FIJI (ImageJ 1.52p Java 1.8.0_172 64 Bit).4 All adjustments to voxel brightness and contrast were kept at constant values within each image set to ensure consistency in the results obtained.

## Results and Discussion

The live-cell fluorescence imaging studies reported herein follow one of two MNM dosing methodologies previously standardized within our prior works.^7,11^ In protocol A, used for studying the impact of MNMs in solution and localized on the cell surface, the nanomachines are loaded into the cell media with imaging and rotor attenuation, being initiated after a 30 min preincubation period, without any subsequent removal. In protocol B (used for investigating the capacity of selected MNMs to cross the phospholipid bilayer), the machines are loaded into the cell media and left to incubate for a set time between 30 min and 24 h, followed by washing the cells three times with fresh MNM-free media, removing any compound left in solution. To investigate the proposed ability of internalizing MNMs to induce apoptotic or other PCD pathways, a slight alteration to protocol B has been introduced. Here, upon 2PE with 710 nm light during imaging, activation times of between 20 s and 5 min are employed instead of the usual constant exposure to prevent disruption of the membrane and subsequent, irreversible, necrotic cell death. These protocols are covered in greater detail in the Experimental Section. In all of the experiments presented within, NIH Swiss mouse embryo fibroblast (NIH 3T3) cells have been used to maintain similarity with all our previously reported studies with untargeted MNM controls. Importantly, all MNMs are used as a stock solution at a standardized 1.00 μM concentration with total dimethyl sulfoxide (**DMSO**, required for MNM solubility) concentration never passing 0.1% in the final cell media to avoid unwanted cell membrane permeabilization. To evaluate any occurrence of necrosis, propidium iodide (**PI**, 0.10 μM) is introduced to the cell media 5 min prior to imaging. PI is nontoxic, noninternalized, intercalating, fluorescent reporter, which remains relatively nonfluorescent in solution due to efficient quenching by water. During necrosis, however, PI can freely cross the disrupted cellular boundaries and intercalate within the cell’s DNA or RNA—displacing the quenching water molecules. This causes an ∼30 nm bathochromic shift in emission wavelength (535 to 565 nm) accompanied by a 10-fold hyperchromic shift, allowing for efficient detection of cell membrane damage and subsequent necrotic cell death.^25^ Crucially, additional visual indicators of differing cell death pathways are also employed by the microscopist to detect and distinguish between necrosis and apoptosis. Necrosis is accompanied by extracellular membrane rupture, blebbing of the cellular membrane, and homogeneous cytosolic autofluorescence, indicating the loss of cellular organelle boundaries, while apoptosis is illustrated by both cell shriveling and loss of focus due to morphological changes and detachment from the microscope slide surface.

Initial experiments, using direct loading and parallel imaging, were undertaken to demonstrate that this family of internalizing MNMs (MNMs 2–4) were able to cause damage to cell membranes, subsequently triggering necrotic cell death, when excited by a 355 nm UV excitation source, which is consistent with our previous reports. This was carried out to ensure that the MNMs used in this study were comparable to those previously evaluated. These initial experiments concluded that under constant 355 nm excitation of the rotor in MNMs 2–4, all MNMs showed irreversible necrotic cell death starting at ∼300 s after initial exposure, consistent with previous results for MNM 1. These results are presented in Supporting Figures S1–S3.

The 2PE cross-section measurements^26–28^ carried out on the core MNM 1 (σ^2^ = 10.3 GM, optical studies shown in Supporting Figure S4)^11^ by way of its weak fluorescence suggest that MNMs 2–4 should also be able to have their rotary motion induced via 710 nm NIR 2PE because of their similar core structures. However, direct measurement of each MNM’s 2PE cross-section remains a challenge due to their significantly lower fluorescence intensity. Based on this hypothesis, a series of 2PE actuation, with PI uptake experiments, were carried out with each MNM 2–4 in solution and compared to the results obtained for MNM 1 in line with protocol A (Figure 2). All of the experiments were executed using a standardized constant photon flux/voxel comparable with previous work (∼2 × 10^29^ cm^–2^ s at 710 nm). These parameters were maintained by carefully pairing and adjusting the objective magnification, numerical aperture (**NA**), laser power, and line scan speed accumulation and averaging. These microscopy studies seemingly confirm that MNMs 2–4 can have rotary motion induced via highly pulsed 710 nm radiation, in the same way as the previously reported, unfunctionalized MNM 1, with the first indications of PI emission from within the nucleus occurring at ∼400 s after initial 2PE, and complete necrotic cell death occurring at or before 750 s–comparable with our previous work.

**Figure 2:**
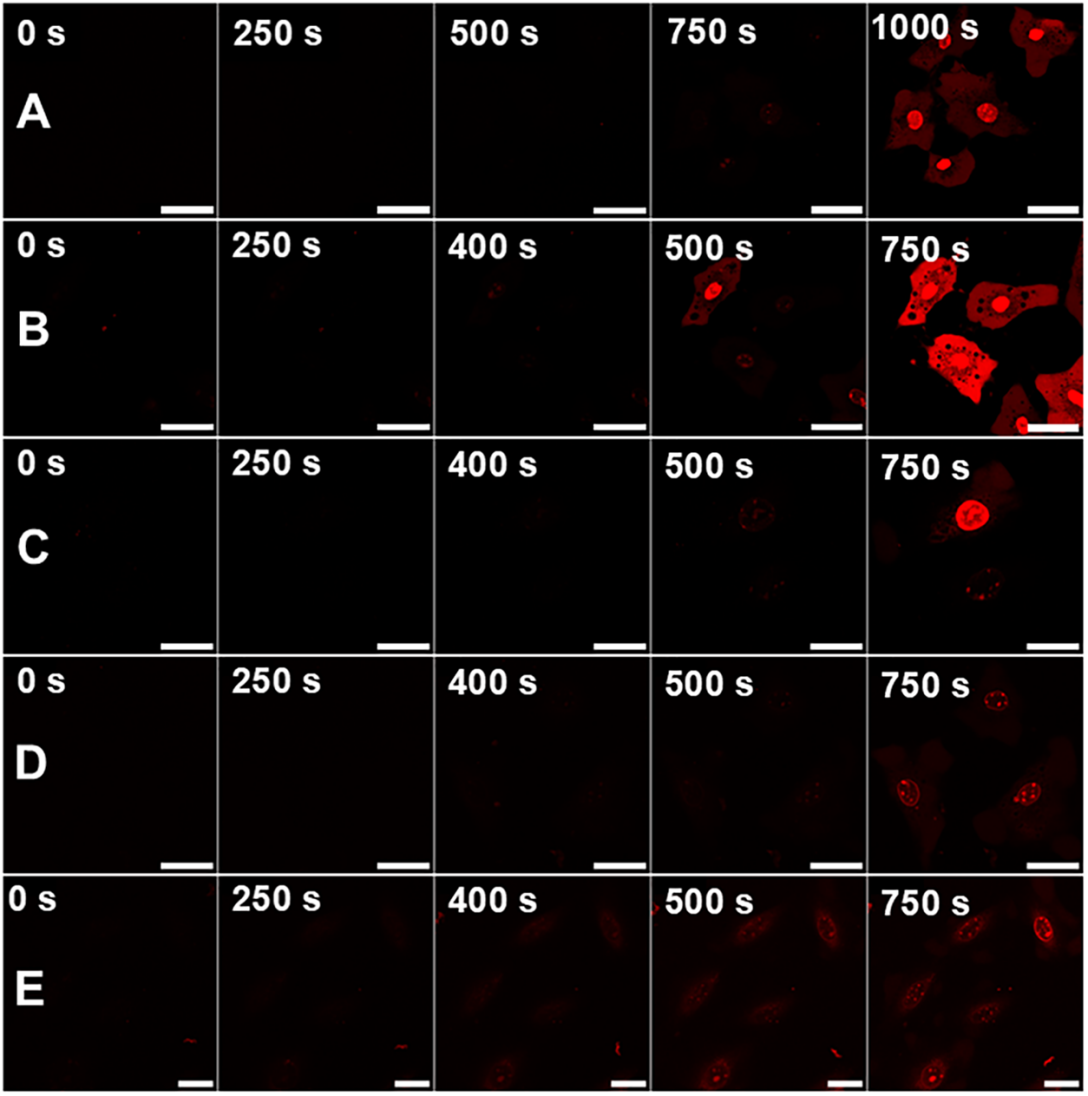
Comparison of MNM ability to induce necrosis in NIH 3T3 cells when activated by 710 nm NIR light for MNMs 1, 2 (PEGylated), and 4 (TPP+ functionalized) quantified by the observation of PI fluorescence within the nucleus. (A) Control sample: NIH 3T3 cells loaded with 100 nM PI and 0.1% DMSO. (B) NIH 3T3 cells loaded with 100 nM PI and 1 μM MNM 1. (C) NIH 3T3 cells loaded with 100 nM PI and 1 μM MNM 2. (D) NIH 3T3 cells loaded with 100 nM PI and 1 μM MNM 3. (E) NIH 3T3 cells loaded with 100 nM PI and 1 μM MNM 4. All image sets collected after 30 min incubation following dosing procedure. Red channel showing detection of PI fluorescence (λ_ex_ = 543 nm, 0.2 mW; λ_em_ = 600–700 nm). All scale bars refer to 25 μm.

Upon successful identification of MNMs 2–4 as viable 2PE candidates for the induction of necrosis, we next used protocol B to determine if any of the proposed functional groups allowed the machines to cross the phospholipid bilayer and internalize within the cell. By washing the samples with MNM-free media prior to imaging and removing any excess MNMs in solution, only those with the capability to cross the cell membrane—or bind to cell surface receptors as illustrated previously^11^—will be left behind to be activated during the subsequent 2PE imaging procedure. Results are presented following 2 h of incubation after MNM addition, followed by fresh MNM-free media washes, as this was deemed to be the minimum incubation time to observe adequate internalization of MNM (Figure 3). However, it should be noted that incubation times between 30 min and 24 h were tested. Shorter incubations provided less apparent changes in Time-To-Necrosis onset (**TTN**) when compared with the control samples, while longer incubations were found unnecessary to show a sufficient increase in internalization compared to the 2 h time frame. Excessive incubation with MNMs also presents a higher likelihood of problems in cell division arising from longer growth on microscope channel slides—please refer to the Supporting Information.

**Figure 3:**
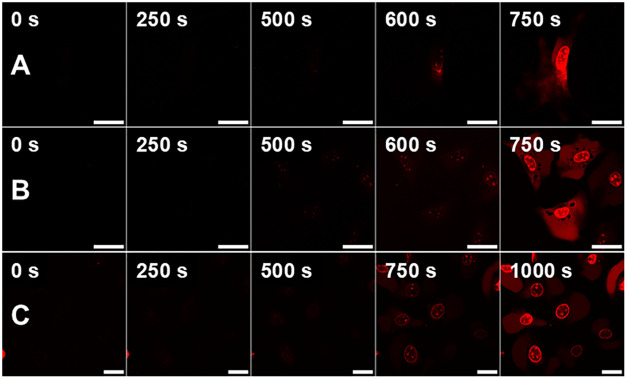
Comparison of internalizing MNM 2, 3, and 4’s ability to cross the cellular membrane after 2 h incubation at 37 °C prior to imaging with excitation with 710 nm NIR light, obtained by measuring the time to irreversible cell death quantified by the observation of PI fluorescence within the nucleus. (A) NIH 3T3 loaded with 1 μM MNM 2, followed by 2 h incubation and washing with MNM-free media 3×, and subsequent staining with 100 nM PI. (B) NIH 3T3 loaded with 1 μM MNM 3, followed by 2 h incubation and washing with MNM-free media 3×, and subsequent staining with 100 nM PI. (C) NIH 3T3 loaded with 1 μM MNM 4, followed by 2 h incubation and washing with MNM-free media 3×, and subsequent staining with 100 nM PI. All image sets collected after 30 min incubation following dosing procedure. Red channel showing detection of PI fluorescence (λ_ex_ = 543 nm, 0.2 mW; λ_em_ = 600–700 nm). All scale bars refer to 25 μm.

Importantly, clearance experiments (described in Supporting Figures S5–S7) were carried out for MNMs 2–4, using protocol B, followed by allowing an additional period for cell division (16–24 h) with further MNM-free media washes before subsequent imaging. This allows for any internalized MNM to be cleared from the cells, most probably via autophagy or other related cellular processes.^29^ All cells dosed with each MNM (1–4), followed by the clearance period with MNM-free media, displayed a return to the control sample (no addition of MNMs) TTN once exposed to NIR light. This provides strong evidence that without activation by either 1PE 355 nm or 2PE 710 nm light, no MNMs possess innate toxicity to the cells studied—with samples being left to replicate for up to 48 h after MNM clearance with no discernible impact on overall cell health as confirmed by cell viability experiments and cell count.

It is clearly shown that MNMs 2–4 can cross, or be lodged within, the phospholipid bilayer, and remain within the cell after complete removal of MNM-containing media. Exhibiting a TTN of between 500 and 600 s, taking longer than protocol A, where MNMs remain in solution but still representing a significant ∼50% reduction when compared with negative control samples. An interesting observation is made in the mitochondrial autofluorescence (λ_ex_ = 355 nm, 20 mW, 400 nJ per voxel; λ_em_ = 440–460 nm) channel when carrying out these microscopy experiments with standard 355 nm 1PE on samples dosed with the TPP+ functionalized MNM 4 (Supporting Figure S8). Here, a difference in the appearance of the mitochondrial network can be seen (Figure 4) when compared to samples containing any other MNM. While showing no observable impact on the viability of the cells studied, these observations imply that the addition of TPP+ moieties enables MNM 4 to localize about the negatively charged mitochondrial membrane within the cells.

**Figure 4:**
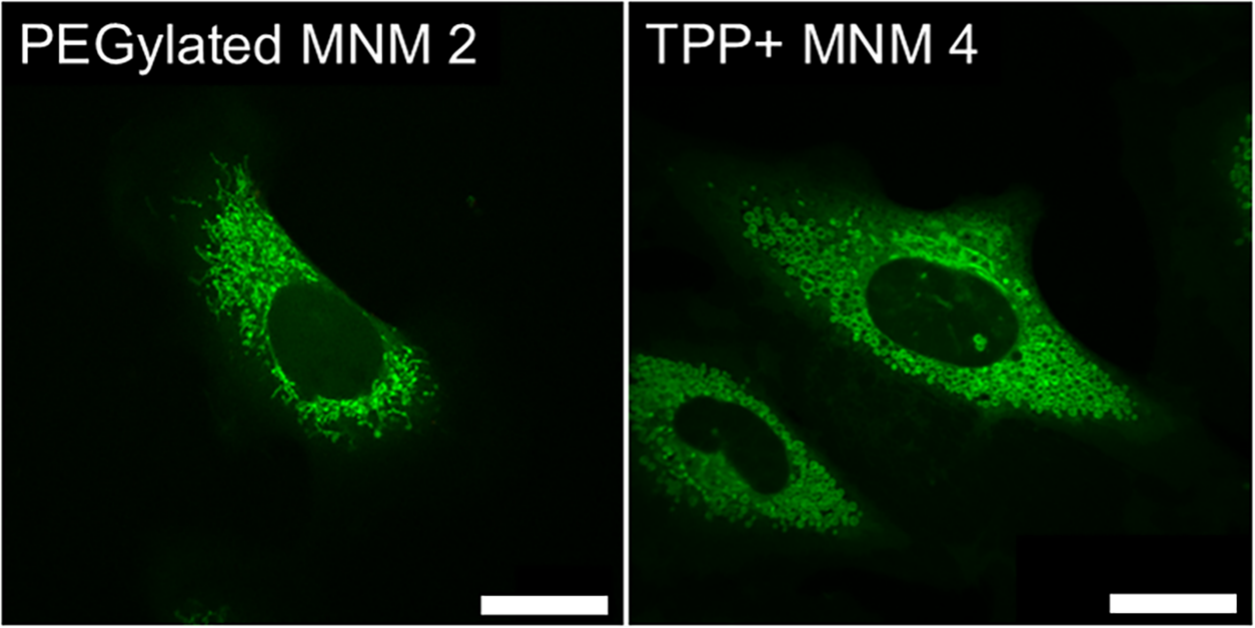
Comparison of mitochondrial autofluorescence profile (λ_ex_ = 355 nm, 20 mW, 400 nJ per voxel; λ_em_ = 440–460 nm) for NIH 3T3 cells dosed with 1 μM PEG functionalized MNM 2 for 30 min before imaging (left) and 1 μM TPP+ functionalized MNM 4 illustrating alterations in mitochondrial network morphology upon TPP+ association. Scale bars 20 μm.

After determining that this new family of MNMs localizes within cells, a new phase of microscopy studies was carried out to understand if these functionalized MNMs can still be activated when internalized. Crucially, this was done to verify if this activation can be timed to induce high enough levels of internal damage to induce clean PCD pathways without high levels of membrane disruption leading to uncontrolled necrosis. TPP+ functionalized MNM 4 was solely selected for these studies due to the higher likelihood of success caused by the association of TPP+ to the negatively charged mitochondrial membrane. In addition, mitochondrial outer membrane permeabilization is widely known to regulate apoptotic cell death, making the mitochondria a commonly sought-after therapeutic target.^30,31^ First, it was necessary to determine the maximum excitation time of the internalized machine that can be administered without leading to the irreversible cascade of necrosis. PI uptake studies were repeated using protocol B, with 2 h incubation times, to allow for complete internalization before excitation. However, exposure to activating wavelengths of light was limited by switching off the relevant laser during the imaging process, followed by extending the imaging window under only 543 nm excitation to observe any PI emission and subsequent membrane rupture and necrotic cell death (Supporting Figure S9). Here, in a similar fashion to the previous experiments, necrosis is induced in the cells with MNM rotor attenuation times as short as 60 s. However, late-stage necrosis due to complete breakdown of the cellular membrane (shown via bright PI emission throughout the internals of the cell, blebbing, and visible holes in the membrane) is observed significantly later than previous investigations. Importantly, it is confirmed that UV exposure times of less than 1 min are inadequate to cause necrotic cell death via the activation of MNM 4 bound to the cells’ mitochondrial network. This provides a window of possible activation times, 0–60 s, where promotion of alternate routes toward cell death may be possible.

To start investigations into this hypothesis, the apoptosis-specific detection dye Annexin V Alexa Fluor 488 conjugate (**AVAF**) is employed—a phospholipid binding protein that binds to phosphatidylserine. In healthy cells, phosphatidylserine is located at the internal cytoplasmic surface of the cellular membrane, making it inaccessible to the non-membrane-permeable annexin V. Cells undergoing apoptosis expose phosphatidylserine to the outer surface of the membrane as a recognition signal for the macrophages responsible for cellular elimination. This can be exploited for fluorescent-based detection methods of apoptosis by attaching numerous emissive conjugates, such as Alexa Fluor 488, to samples of annexin V.^32^ Initial controls were carried out on both an MNM-free control sample, as well as a sample dosed with unfunctionalized MNM 1 in solution with 100 nM AVAF. This ensures that the inclusion of the AVAF stain does not have any measurable impact on the TTN caused by MNM rotor activation (Supporting Figure S10). A good agreement is found between these results and those previously reported. Interestingly, AVAF emission is detected during the final frames of these experiments, where the cell is exhibiting late-stage necrotic death. When undergoing necrosis, perforations in the cell’s outer membrane are formed, allowing PI to enter the cell and bind to DNA, indicating necrotic cell death. However, this will of course also allow for AVAF to also enter the internal cellular space, binding to the phosphatidylserine molecules held on the inner surface of the phospholipid bilayer and subsequently exhibiting a 100-fold increase in fluorescence intensity. This illustrates the importance of costaining with a necrosis-specific detection reagent such as PI. Thus, an important distinction should be made that it is not only detectable AVAF emission that indicates apoptosis in a sample but AVAF emission specifically unaccompanied by red PI emission, with additional confirmation provided by the microscopic observation of typical apoptotic morphology changes, such as cell shriveling and rounding. This ensures that no membrane disruption has occurred, and thus no necrotic cell death has taken place.

After confirming that the addition of AVAF to the samples under investigation has little to no measurable impact on any previously established results, experimental protocol B was repeated on NIH 3T3 samples dosed with MNM 4 with an incubation time of 2 h. However, MNM excitation was limited to the first 20–60 s of the protocol. While imaging using the 543 nm PI and 488 nm AVAF channels continued to observe any indication of cell death (Figure 5).

**Figure 5:**
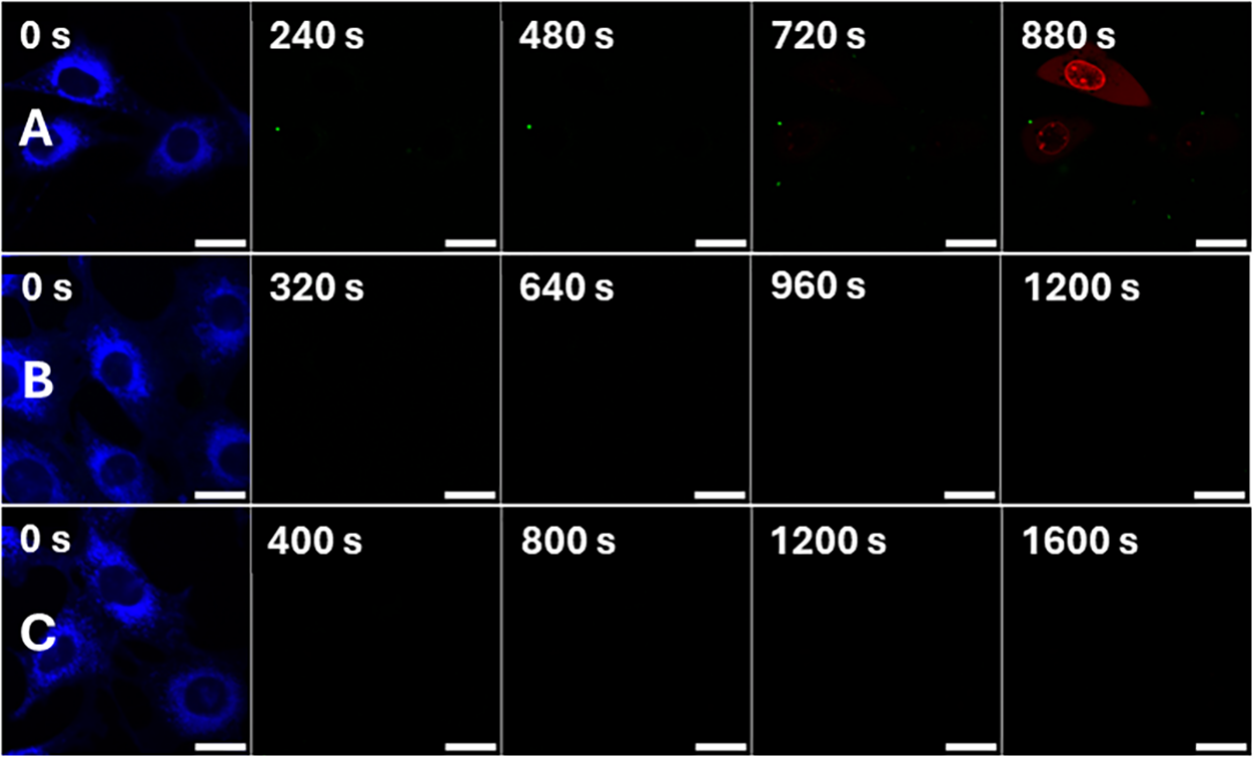
Microscopic observation of cell death caused by excitation of internalized MNM 4 (TPP+ functionalized, 2 h incubation with MNM 4 in solution before washing) at 355 nm for varying periods of rotor activation (1 min, 30 s, 20 s). Total experiment run times are shown for each image. (A) NIH 3T3 cells loaded with 0.5 μM MNM 4 followed by 2 h incubation and washing with MNM-free media, and subsequent staining with 100 nM PI, 100 nM AV, and 0.1% DMSO. Exposed to 355 nm UV laser light for 1 min. (B) NIH 3T3 cells loaded with 0.5 μM MNM 4 followed by 2 h incubation and washing with MNM-free media, and subsequent staining with 100 nM PI, 100 nM AV, and 0.1% DMSO. Exposed to 355 nm UV laser light for 30 s. (C) NIH 3T3 cells loaded with 0.5 μM MNM 4 followed by 2 h incubation and washing with MNM-free media, and subsequent staining with 100 nM PI, 100 nM AVAF, and 0.1% DMSO. Exposed to 355 nm UV laser light for 20 s. All image sets were collected after 30 min incubation following staining procedure. Overlaid channels of PI fluorescence (Red, λ_ex_ = 543 nm, 0.2 mW; λ_em_ = 600–700 nm), AVAF fluorescence (Green, λ_ex_ = 488 nm, 0.2 mW; λ_em_ = 500–550 nm), and mitochondrial autofluorescence (Blue, λ_ex_ = 355 nm, 20 mW, 400 nJ per voxel; λ_em_ = 440–460 nm). All scale bars refer to 20 μm.

As in previous control experiments, where the maximum length of excitation tolerable before irreversible necrosis onset was determined, 60 s of rotor excitation caused sufficient nanomechanical damage to induce necrosis in the cells studied. This rotor excitation time was reduced first to 30 s, followed by 20 s (Figure 5B,C, respectively). While this failed to cause any visible onset of necrosis, it also seems not to show any AVAF emission from the outer membrane of the cell. Initially implying that no form of cell death has occurred. Seemingly concluding that activation of the internalized MNM 4 caused insufficient damage to trigger any PCD pathways. However, surprisingly different conclusions can be drawn from studying the transmission images presented in Figure 6.

**Figure 6:**
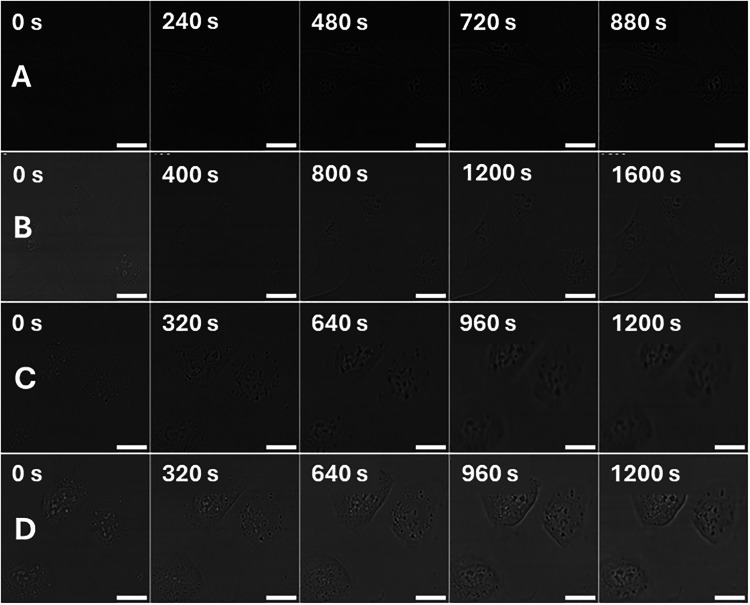
Microscopic observation of cell death caused by excitation of internalized MNM 4 (TPP+ functionalized) at 355 nm for varying periods of rotor activation (1 min, 30 s, 20 s) visualized by morphological changes observed in the transmission image. (A) NIH 3T3 cells loaded with 0.5 μM MNM 4 followed by 2 h incubation and washing with MNM-free media, and subsequent staining with 100 nM PI, 100 nM AV, and 0.1% DMSO. Exposed to 355 nm UV laser light for 1 min. (B) NIH 3T3 cells loaded with 0.5 μM MNM 4 followed by 2 h incubation and washing with MNM-free media, and subsequent staining with 100 nM PI, 100 nM AV, and 0.1% DMSO. Exposed to 355 nm UV laser light for 20 s. (C, D) NIH 3T3 cells loaded with 0.5 μM MNM 4 followed by 2 h incubation and washing with MNM-free media, and subsequent staining with 100 nM PI, 100 nM AV, and 0.1% DMSO. Exposed to 355 nm UV laser light for 30 s. All image sets collected after 30 min incubation following staining procedure. All scale bars refer to 20 μm.

When exposed to excitation/activation light of the MNM’s rotor for 60 s, additional confirmation of the previously established necrotic cell death is seen in the transmission images (Figure 6) with bulging of the cellular boundaries and blebbing. In contrast, very little change in morphology is seen when internalized MNM 4 is only activated for 20 s, implying cell health was maintained—an expected result based upon the previous findings of no emission in the fluorescent channels for either PI (650 nm) or AVAF (525 nm) (Figure 5). Interesting results are seen, however, for the two experiments with 30 s of laser uptime. In these cases, dramatic changes are seen in cell morphology when studying the transmission images in Figure 6; the cell boundaries clearly shrivel up toward the center of the body, accompanied by a significant loss of focus, implying the cell has detached from the slide surface and is now freely moving in solution. Crucially, these observations are coupled with a complete lack of emission with the PI fluorescence channel in Figure 5B,C. Despite the lack of signal acquired from the AV channel, these morphological observations imply that a non-necrotic cell death pathway has been accessed by the activation of the internalized MNM 4. The debated reliability of AVAF to consistently detect apoptotic cell death is reported elsewhere and helps to rationalize this difference as a result.^33^ However, the absence of 500–550 nm AVAF emission is also rationalized by several possible explanations. First, the release of the studied cell from the slide surface and into solution (a key indication of apoptosis and other PCD pathways) caused a loss of focus in the system, which may inhibit the detection of any less intense signal in the 550 nm channel. Additionally, the time scale of the experiments may not be long enough for the cells studied to shuttle their phosphatidylserine to the outer surface of the membrane. Thus, there is no binding site for the AV molecules and therefore no detectable emission. Finally, there are many additional PCD pathways that may be triggered by nanomechanical damage to the mitochondria that do not involve the shuttering of phosphatidylserine that could be observed.^34^

Of course, there are many biomarkers of different PCD pathways with numerous existing protocols for their detection and measurement—both within a cell live imaging context and using addition technologies. In addition to the externalized phosphatidylserine, which acts as the binding partner for AVAF, common targets include activated caspases, cytochrome c, p-53, cytokeratins, and nucleosomal DNA. Of particular interest to this work are methods that may be able to further probe, and distinguish between, differing modes of PCD with better accuracy than AVAF. Methods focusing on mitochondrial damage/alteration are particularly appealing, such as the 3-(4,5-dimethylthiazol-2-yl)-2,5-diphenyltetrazolium bromide (MTT) assay, where added MTT is converted to a formazan product with clearly apparent absorbance at 590 nm dependent on mitochondrial activity—meaning cells with damaged mitochondria show little to no absorbance. In addition to this, cytochrome c detection can be incredibly helpful for distinguishing apoptosis from other forms of PCD. During apoptosis, cytochrome c is translocated from the mitochondria to the cytosol, interacting with APAF1, triggering the caspase cascade and formation of apoptosomes. Anticytochrome c monoclonal antibodies tagged with dyes such as Alexa 488 can be used to image the start of this process, while caspase-specific detection stains can detect the later stages of the process.^35^ A thorough investigation using multiple of these methods may allow for greater knowledge of the specific mode of PCD observed in this work, compared to AVAF alone, and provides a very exciting avenue for follow-up studies.

Importantly, MNM 4, if left unactivated, was found to efficiently clear from within the cell after one period of cell division—despite the clear evidence of mitochondrial association and ability to induce both necrotic and non-necrotic cell death (Figure 7). Here, NIH 3T3 samples dosed with 1 μM MNM 4, left to incubate for 2 h, washed with MNM-free media, allowed to divide for 16 h, and washed again with MNM-free media, resulting in an increase in TTN to levels congruent with control samples never exposed to MNMs. Activation with 355 nm UV after 2 h of incubation with MNM 4 without any clearance time resulted in accelerated necrosis—in line with previously described experiments. These exciting results bolster the possibility of this technology leading to new, type IV, PDT. With cells unexposed to the activation wavelengths showing no measurable negative outcome due to MNM internalization, implying any MNM-exposed tissue not targeted by the therapeutic light would experience no adverse side effects of the treatment.

**Figure 7:**
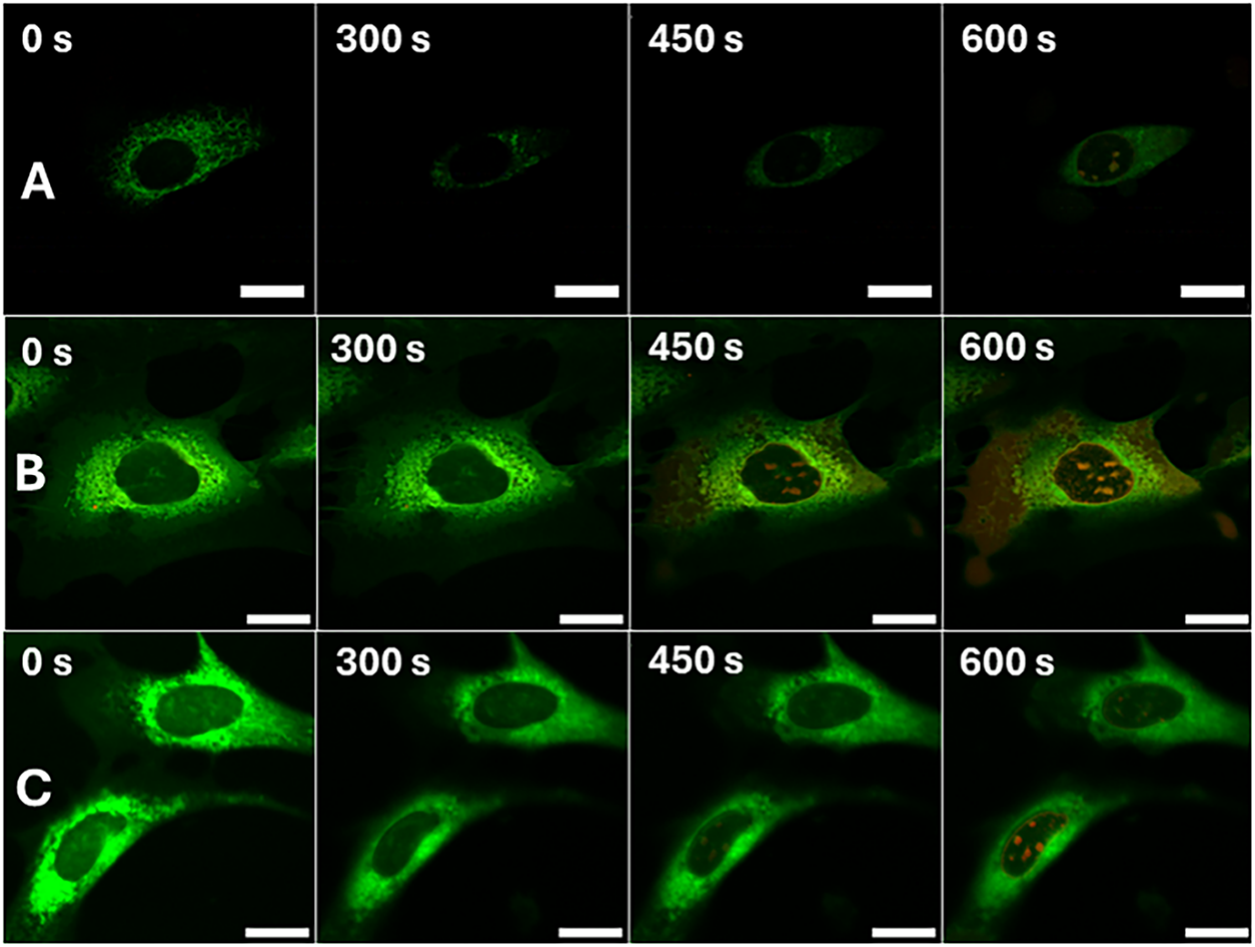
Microscopic observation of cell death caused by excitation of internalized MNM 4 (TPP+ functionalized) at 355 nm, comparing a control sample loaded with no MNM, internalized MNM 4, and a sample left for a 16 h clearance procedure with no MNM 4 still within the system (UV exposure times are shown for each image), quantified by the observation of PI fluorescence within the nucleus. (A) Control sample: NIH 3T3 cells were loaded with 100 nM PI and 0.1% DMSO. (B) NIH 3T3 loaded with 0.5 μM MNM 4, followed by 2 h incubation and washing with MNM-free media, and subsequent staining with 100 nM PI. (C) NIH 3T3 loaded with 0.5 μM MNM 4, followed by 2 h incubation, washing with MNM-free media, 16 h clearance, and subsequent staining with 100 nM PI. All image sets collected after 30 min incubation following staining procedure. Overlaid channels of PI fluorescence (λ_ex_ = 543 nm, 0.2 mW; λ_em_ = 600–700 nm), and mitochondrial autofluorescence (λ_ex_ = 355 nm, 20 mW, 400 nJ per voxel; λ_em_ = 440–460 nm). All scale bars refer to 20 μm.

## Conclusions

Using a new family of internalizing MNMs functionalized with PEG and TPP+ moieties, alongside both traditional 355 nm UV and 2PE 710 nm NIR rotor attenuation, we present significant leaps in the development of MNM-based type IV PDTs for cell-specific elimination. Both functionalizations were evaluated for their ability to passively cross the outer membrane of NIH 3T3 cells. Good levels of passive uptake are seen for both families of MNMs with incubation times as short as 30 min, measured by the acceleration of the necrosis onset in samples with no nanomachines left in solution. This was observed by the rapid increase in the brightness of PI emission from within the nucleus of the cells under study. While both systems of functionalized MNM (PEG and TPP+) can efficiently cross the phospholipid bilayer and be activated from within the cell, evidence is presented that TPP+ functionalized species possess an additional ability to localize at the mitochondrial membrane. This was observed by morphological changes within the 355 nm UV-induced autofluorescence of the cell mitochondrial network. This is thought to be caused by electrostatic attraction between the diffuse positive charge across the TPP+ moieties’ phenyl system and the negative potential of the mitochondrial envelope. This represents a possibility of activating these MNMs from within the cell, causing irreversible nanomechanical damage, without disrupting the outer membrane, leading to possible induction of non-necrotic cell death.

PI/AVAF staining of cellular samples with internalized, mitochondrially bound, TPP+ functionalized MNM 4 was carried out to evaluate the possibility of inducing apoptotic cell death via MNM-based type IV PDT. While these procedures did not elicit AVAF emission via binding to surface shuttled phosphatidylserine, interesting morphological changes were observed in the transmission channel, posing some noteworthy conclusions. With a rotor excitation of 30 s, internalized TPP+ functionalized MNM 4 consistently exhibited typical PCD alterations in cell morphology, namely, cell shrinkage and release into solution from the slide surface. This indicates that the internal nanomechanical damage has either induced a nonapoptotic PCD pathway without phosphatidylserine shuttling or is undergoing apoptotic cell death, but limitations in the AVAF-based detection system prohibit emission from being observed. This provides avenues for further inquiry into distinguishing between different mechanisms of cell death in live-cell microscopy systems.

While the in vivo activation of the internalized MNMs presented here is incredibly exciting for possible future application, it is important to consider translational problems when extending this technology toward medicinal applications, and importantly, the often overstated penetration depth of multiphoton activation signals. While it is true that the shift toward lower-energy wavelengths, such as 710 nm, does indeed allow for greater levels of tissue penetration when compared to our previously utilized 355 nm UV light source, the scattering of this light will lead to less efficient attenuation of the MNM rotor, as well as a reduction in inherent confocality. While possible to be extended to surface-level cancers in the immediate term, deeper applications are still an avenue where further work is required. In addition to this, as with any proposed drug candidate, metabolite studies are also an important next step, both to measure and improve pharmacokinetic and pharmacodynamic properties, as well as to understand the safety of any determined metabolic products.

By using the established experimental methods of this work to introduce non-necrotic cell death by way of internal cellular activation of MNMs with two-photon activation, biologically favorable cell death can be achieved using less phototoxic wavelengths of light. MNM-based treatment methods may represent a multiuse therapeutic with the ability to induce multiple modes of cell death by simple tweaking of excitation conditions depending on the specific needs of the patient, or disease, being treated.

## Supplementary Material



## Data Availability

All data generated and analyzed during this study including spectra and drawings are available from the corresponding author upon request. Custom codes written and developed and used during this study are available from the corresponding author upon request on a collaboration basis.

## Notes

Microscopy results (Figures S1–S10); methods for the synthesis of MNMs 1–4 and all intermediates alongside relevant characterization data (Schemes S1–S8) (PDF)
