# Supplementary material for: Programmed Cell Death via Type IV Photodynamic Therapy Using Internalized Two-Photon Activated Molecular Nanomachines
Source: ACS Appl Bio Mater. 2025 Oct 13;8(11):9982–92. doi: 10.1021/acsabm.5c01318 (PMC12628324; doi:10.1021/acsabm.5c01318)
Supplement: Supplementary file 1 [file mt5c01318_si_001.pdf]

Supporting Information

Programmed Cell Death via Type IV Photodynamic Therapy Using Internalised Two-Photon Activated Molecular Nanomachines

Authors: Thomas. S. Bradford[1], Dongdong Liu[2], James M. Tour[3], and Robert Pal[1]*.

1. Department of Chemistry
   Durham University
   South Road, Durham
   DH1 3LE, United Kingdom

2. Department of Chemistry
   Rice University
   Houston Texas 77005, United States

3. Department of Chemistry
   Department of Materials Science and NanoEngineering
   Rice Advanced Materials Institute and Smalley-Curl Institute
   NanoCarbon Center
   Rice University
   Houston Texas 77005, United States

Corresponding author email: robert.pal@durham.ac.uk

Supplementary Figures

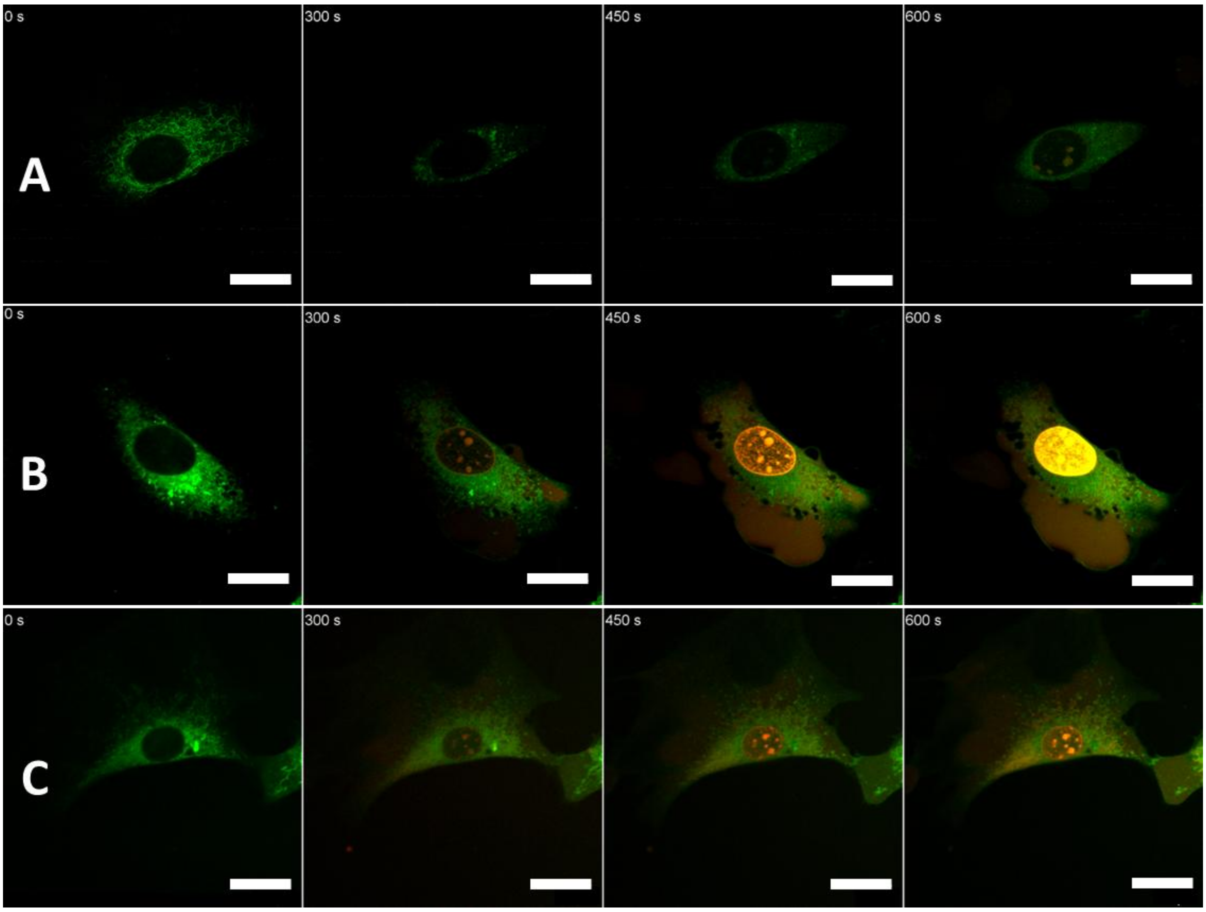

Figure S1 Microscopic observation of necrotic cell death caused by excitation at 355 nm (UV-exposure times are shown for each image), quantified by the observation of PI fluorescence within the nucleus. **A** – Control sample: NIH 3T3 cells loaded with 100 nM PI and 0.1% DMSO. **B** – NIH 3T3 cells loaded with 100 nM PI and 0.5 μM **MNM 1**. **C** - NIH 3T3 cells loaded with 100 nM PI and 0.5 μM **MNM 2**. All image sets collected after 30 minutes incubation after dosing procedure. Overlaid channels of PI fluorescence (λ_{ex} = 543 nm, 0.2 mW; λ_{em} = 600-700 nm), and mitochondrial autofluorescence (λ_{ex} = 355 nm, 20 mW, 400 nJ per voxel; λ_{em} = 440-460 nm). All scale bars refer to 20 μm.

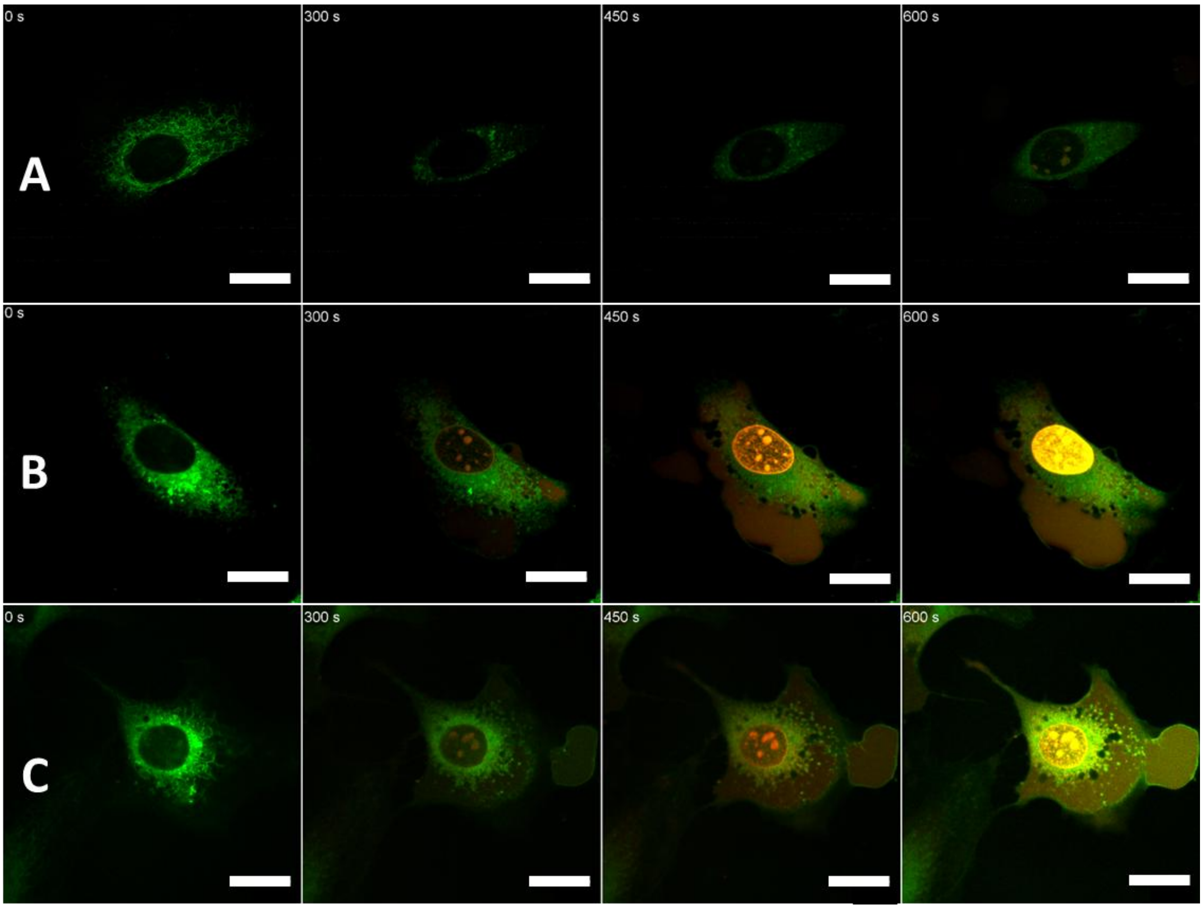

Figure S2 Microscopic observation of necrotic cell death caused by excitation at 355 nm (UV-exposure times are shown for each image), quantified by the observation of PI fluorescence within the nucleus. **A** – Control sample: NIH 3T3 cells loaded with 100 nM PI and 0.1% DMSO. **B** – NIH 3T3 cells loaded with 100 nM PI and 0.5 µM **MNM 1**. **C** - NIH 3T3 cells loaded with 100 nM PI and 0.5 µM **MNM 3**. All image sets collected after 30 minutes incubation after dosing procedure. Overlaid channels of PI fluorescence (λ_{ex} = 543 nm, 0.2 mW; λ_{em} = 600-700 nm), and mitochondrial autofluorescence (λ_{ex} = 355 nm, 20 mW, 400 nJ per voxel; λ_{em} = 440-460 nm). All scale bars refer to 20 µm.

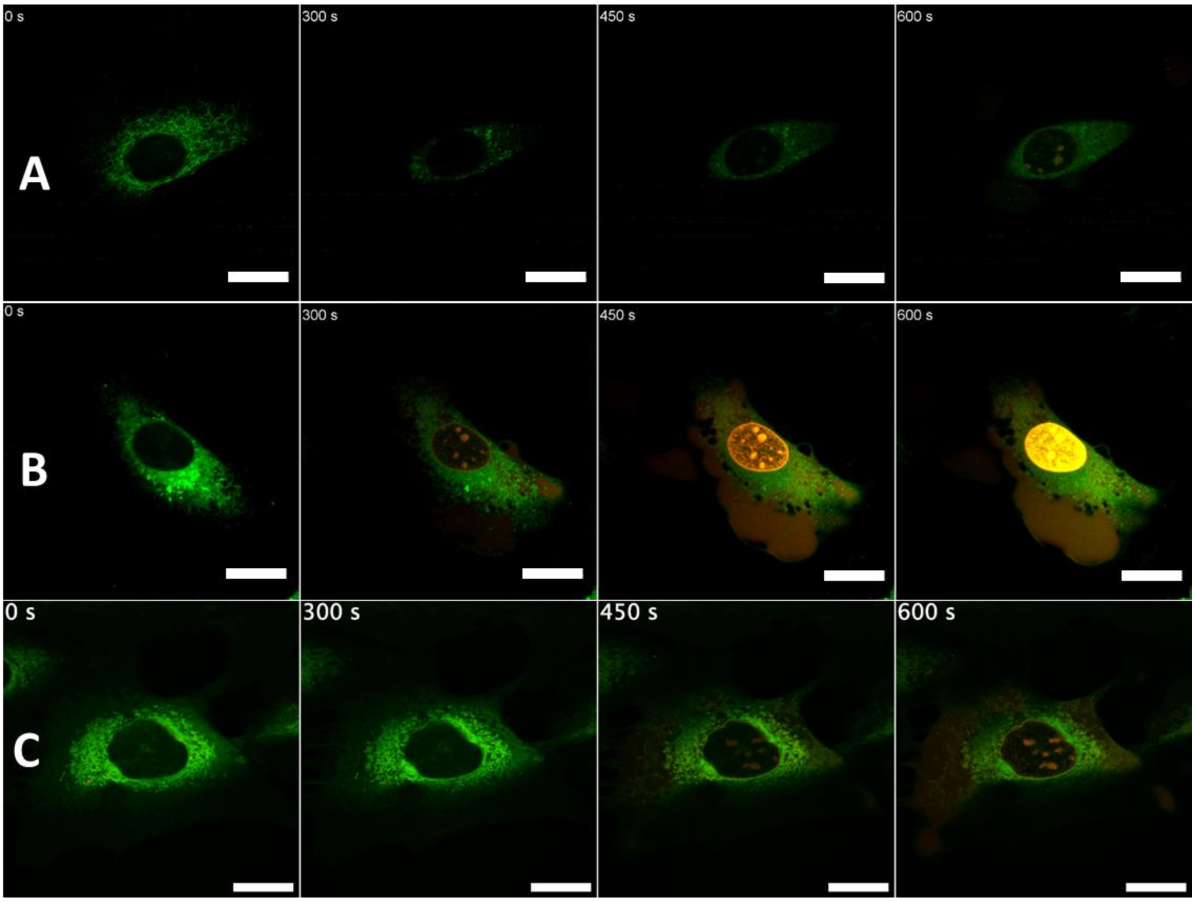

Figure S3 Microscopic observation of necrotic cell death caused by excitation at 355 nm (UV-exposure times are shown for each image), quantified by the observation of PI fluorescence within the nucleus. **A** – Control sample: NIH 3T3 cells loaded with 100 nM PI and 0.1% DMSO. **B** – NIH 3T3 cells loaded with 100 nM PI and 0.5 µM **MNM 1**. **C** - NIH 3T3 cells loaded with 100 nM PI and 0.5 µM **MNM 4**. All image sets collected after 30 minutes incubation after dosing procedure. Overlaid channels of PI fluorescence (λ_{ex} = 543 nm, 0.2 mW; λ_{em} = 600-700 nm), and mitochondrial autofluorescence (λ_{ex} = 355 nm, 20 mW, 400 nJ per voxel; λ_{em} = 440-460 nm). All scale bars refer to 20 µm.

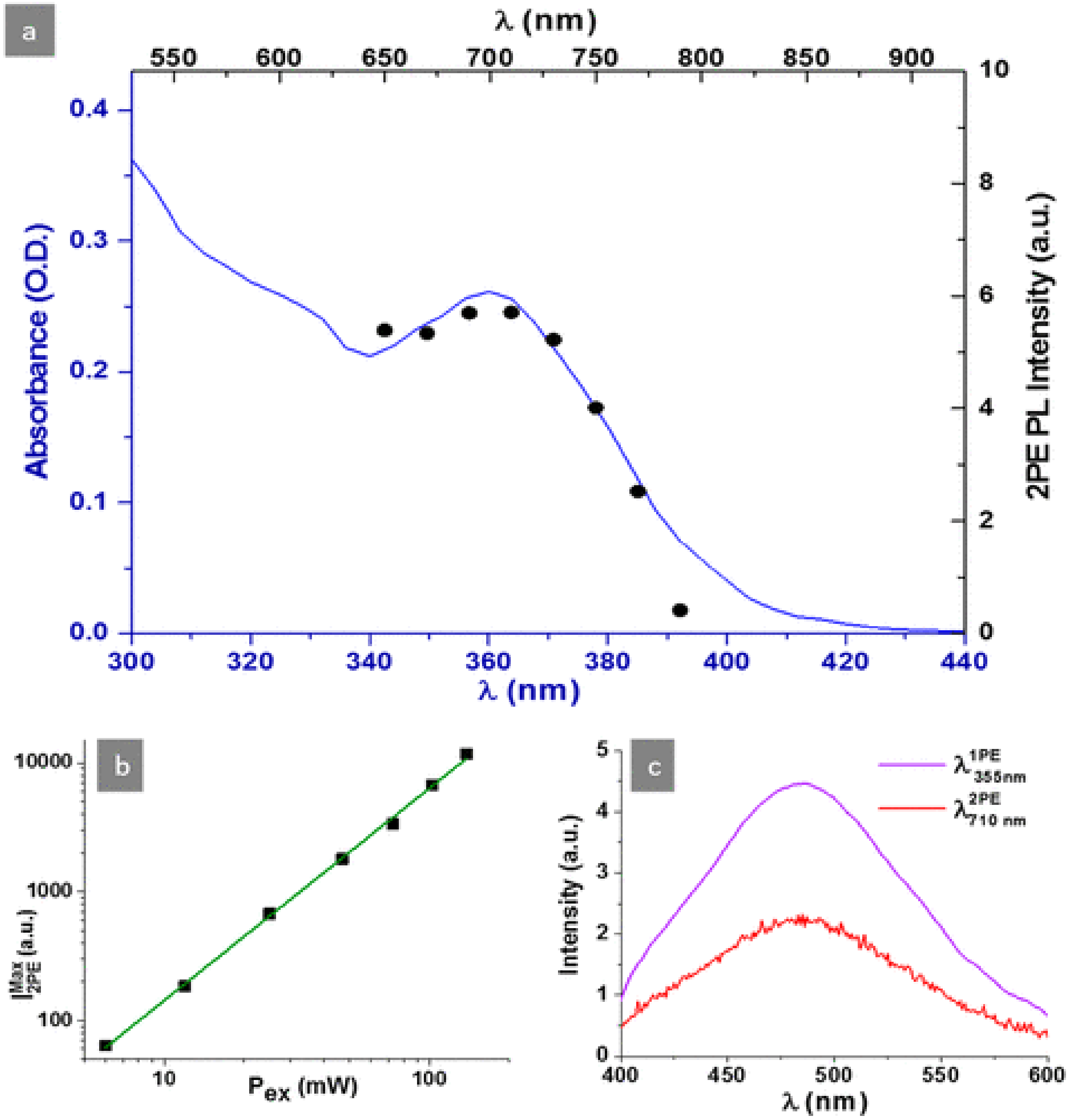

Figure S4 Optical studies to assess 2PE NIR excitation efficacy of MNM 1. (a) One photon absorption (blue) and two (●) photon excitation spectrum (λ_{em} = 500 nm) plot of **1** (Φ_f = 3.7 × 10^{-3}, Σ = 15400 dm^3 mol^{-1} cm^{-1}). (b) Excitation power dependency (green, ■) of the PL intensity (10^{-50} cm^4·s $photon^{-1}$), slope 1.96 ± 0.1 in DMSO. (c) 1PE (purple, λ_{ex} = 355 nm) and 2PE (red, λ_{ex} = 710 nm) emission spectrum of MNM 1.[1]

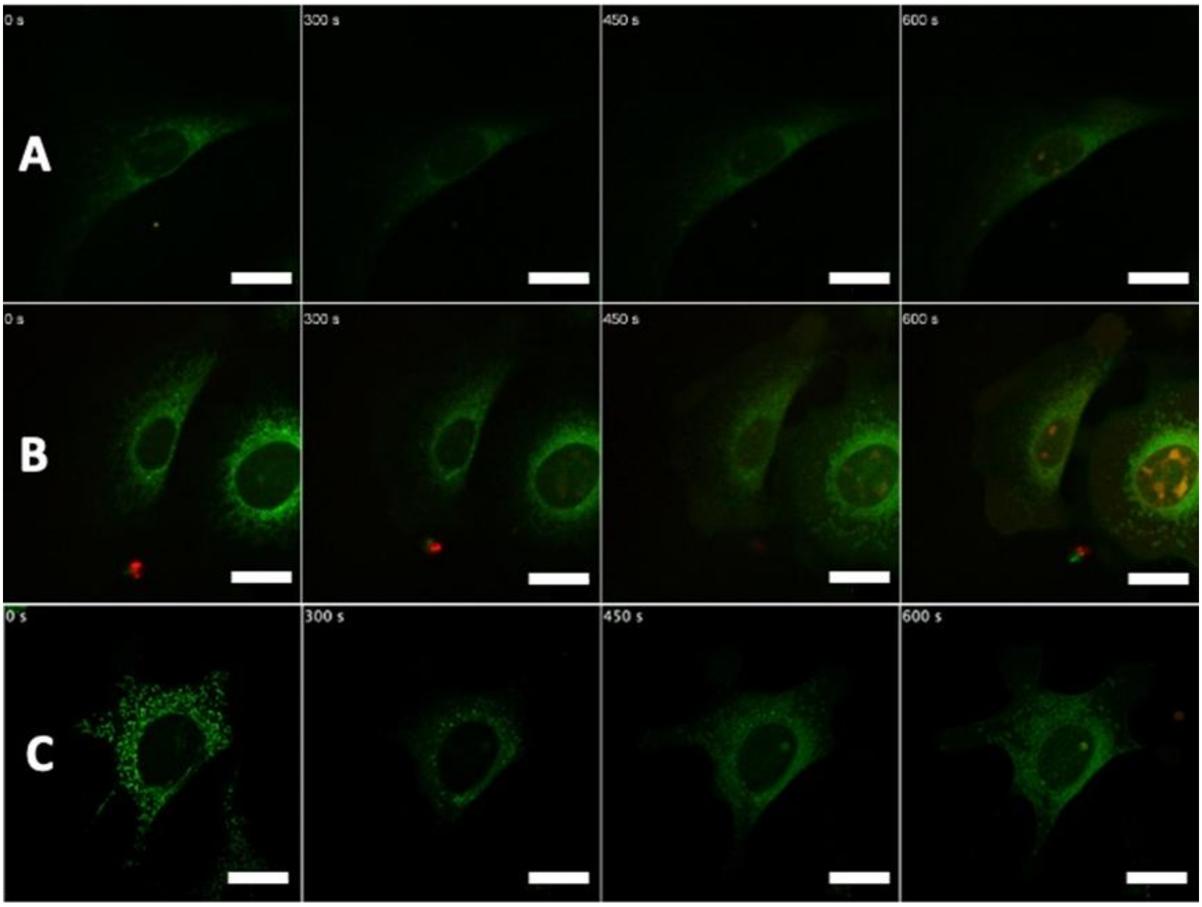

Figure S5 Microscopic observation of cell death caused by excitation at 355 nm (UV-exposure times are shown for each image), quantified by the observation of PI fluorescence within the nucleus. **A** – NIH 3T3 loaded with 0.5 μM **MNM 2**, followed by 30 minutes incubation and washing with MNM free media, and subsequent staining with 100 nM PI. **B** – NIH 3T3 loaded with 0.5 μM **MNM 2**, followed by 2-hour incubation and washing with MNM free media, and subsequent staining with 100 nM PI. **C** - NIH 3T3 loaded with 0.5 μM **MNM 2**, followed by 2-hour incubation, washing with MNM free media, 16-hour clearance, and subsequent staining with 100 nM PI. All image sets collected after 30 minutes incubation after staining procedure. Overlaid channels of PI fluorescence (λ_{ex} = 543 nm, 0.2 mW; λ_{em} = 600-700 nm), and mitochondrial autofluorescence (λ_{ex} = 355 nm, 20 mW, 400 nJ per voxel; λ_{em} = 440-460 nm). All scale bars refer to 20 μm.

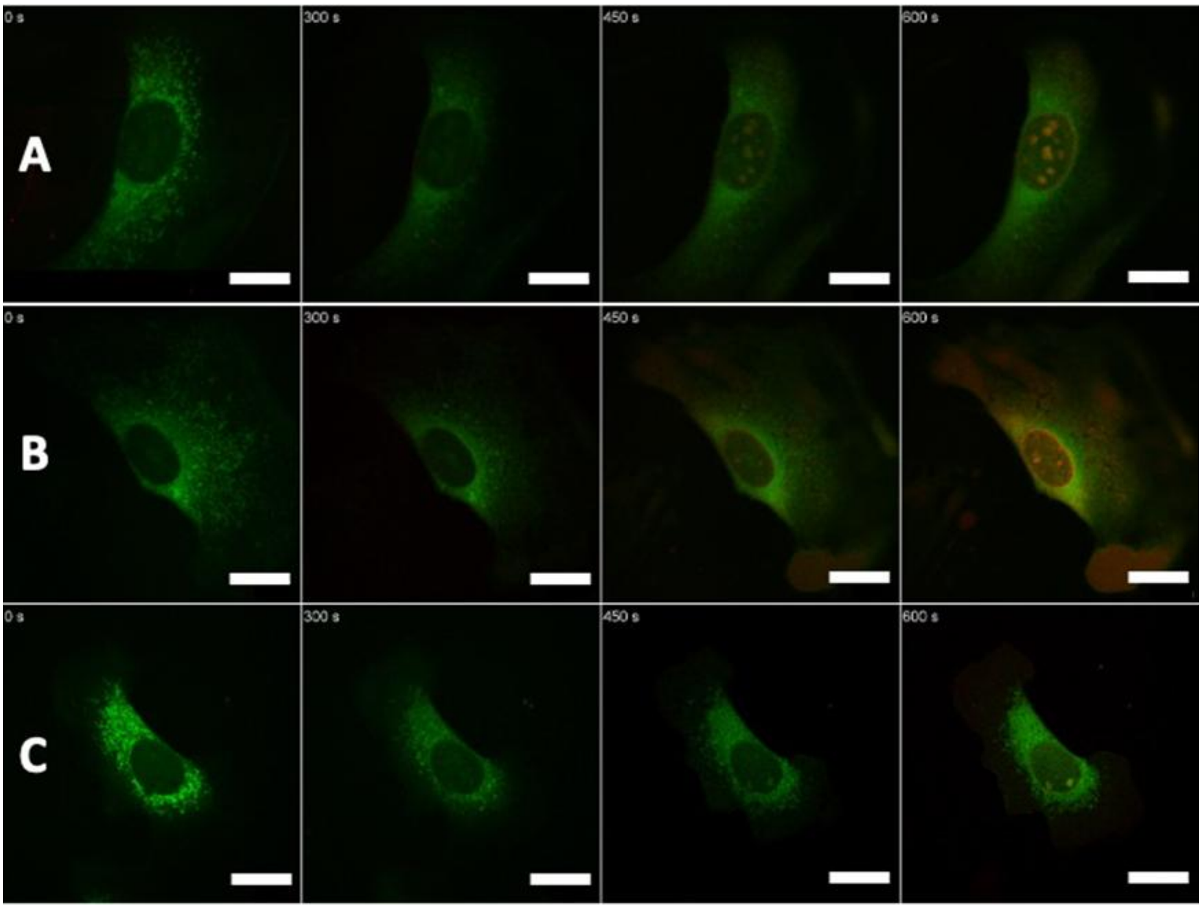

Figure S6 Microscopic observation of cell death caused by excitation at 355 nm (UV-exposure times are shown for each image), quantified by the observation of PI fluorescence within the nucleus. **A** – NIH 3T3 loaded with 0.5 μM **MNM 3**, followed by 30 minutes incubation and washing with MNM free media, and subsequent staining with 100 nM PI. **B** – NIH 3T3 loaded with 0.5 μM **MNM 3**, followed by 2-hour incubation and washing with MNM free media, and subsequent staining with 100 nM PI. **C** - NIH 3T3 loaded with 0.5 μM **MNM 3**, followed by 2-hour incubation, washing with MNM free media, 16-hour clearance, and subsequent staining with 100 nM PI. All image sets collected after 30 minutes incubation after staining procedure. Overlaid channels of PI fluorescence (λ_{ex} = 543 nm, 0.2 mW; λ_{em} = 600-700 nm), and mitochondrial autofluorescence (λ_{ex} = 355 nm, 20 mW, 400 nJ per voxel; λ_{em} = 440-460 nm). All scale bars refer to 20 μm.

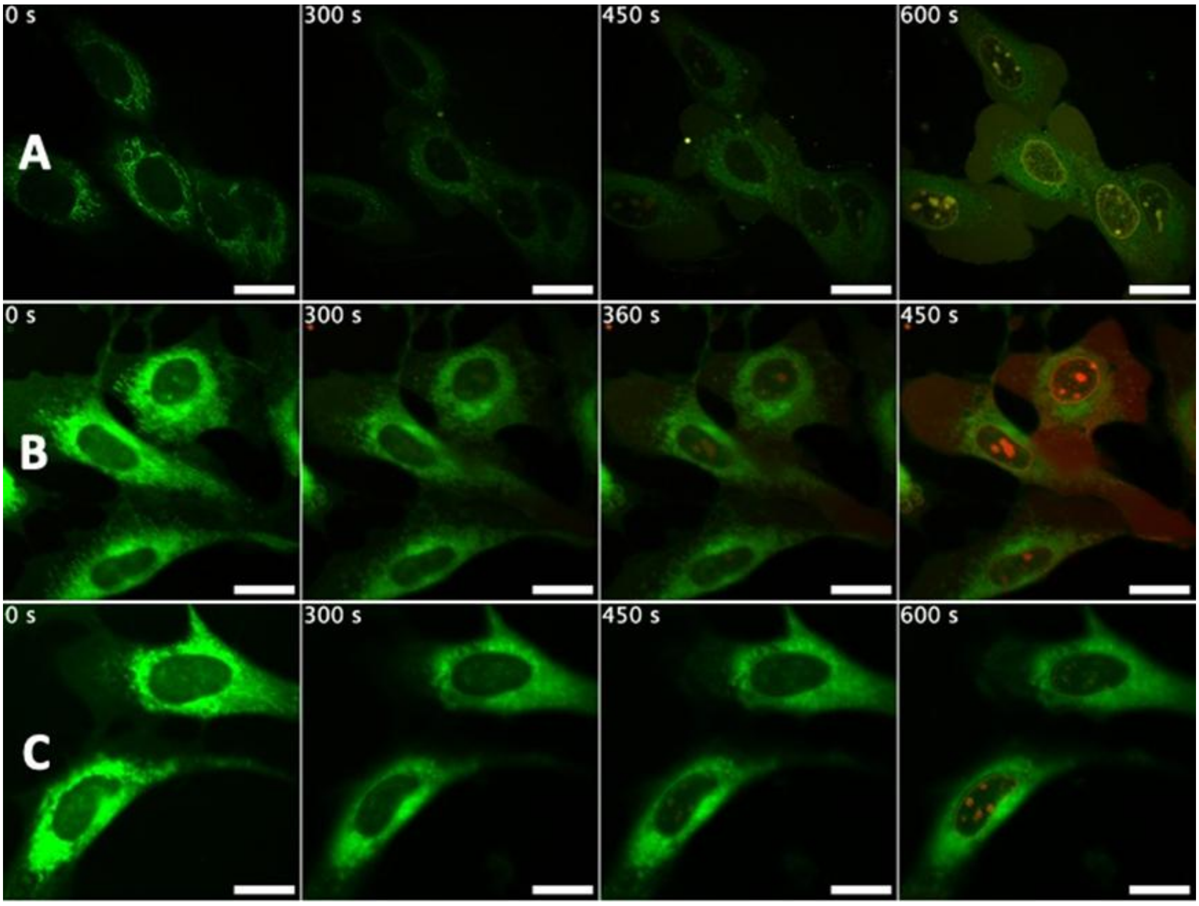

Figure S7 Microscopic observation of cell death caused by excitation at 355 nm (UV-exposure times are shown for each image), quantified by the observation of PI fluorescence within the nucleus. **A** – NIH 3T3 loaded with 0.5 μM **MNM 4**, followed by 30 minutes incubation and washing with MNM free media, and subsequent staining with 100 nM PI. **B** – NIH 3T3 loaded with 0.5 μM **MNM 4**, followed by 2-hour incubation and washing with MNM free media, and subsequent staining with 100 nM PI. **C** - NIH 3T3 loaded with 0.5 μM **MNM 4**, followed by 2-hour incubation, washing with MNM free media, 16-hour clearance, and subsequent staining with 100 nM PI. All image sets collected after 30 minutes incubation after staining procedure. Overlaid channels of PI fluorescence (λ_{ex} = 543 nm, 0.2 mW; λ_{em} = 600-700 nm), and mitochondrial autofluorescence (λ_{ex} = 355 nm, 20 mW, 400 nJ per voxel; λ_{em} = 440-460 nm). All scale bars refer to 20 μm.

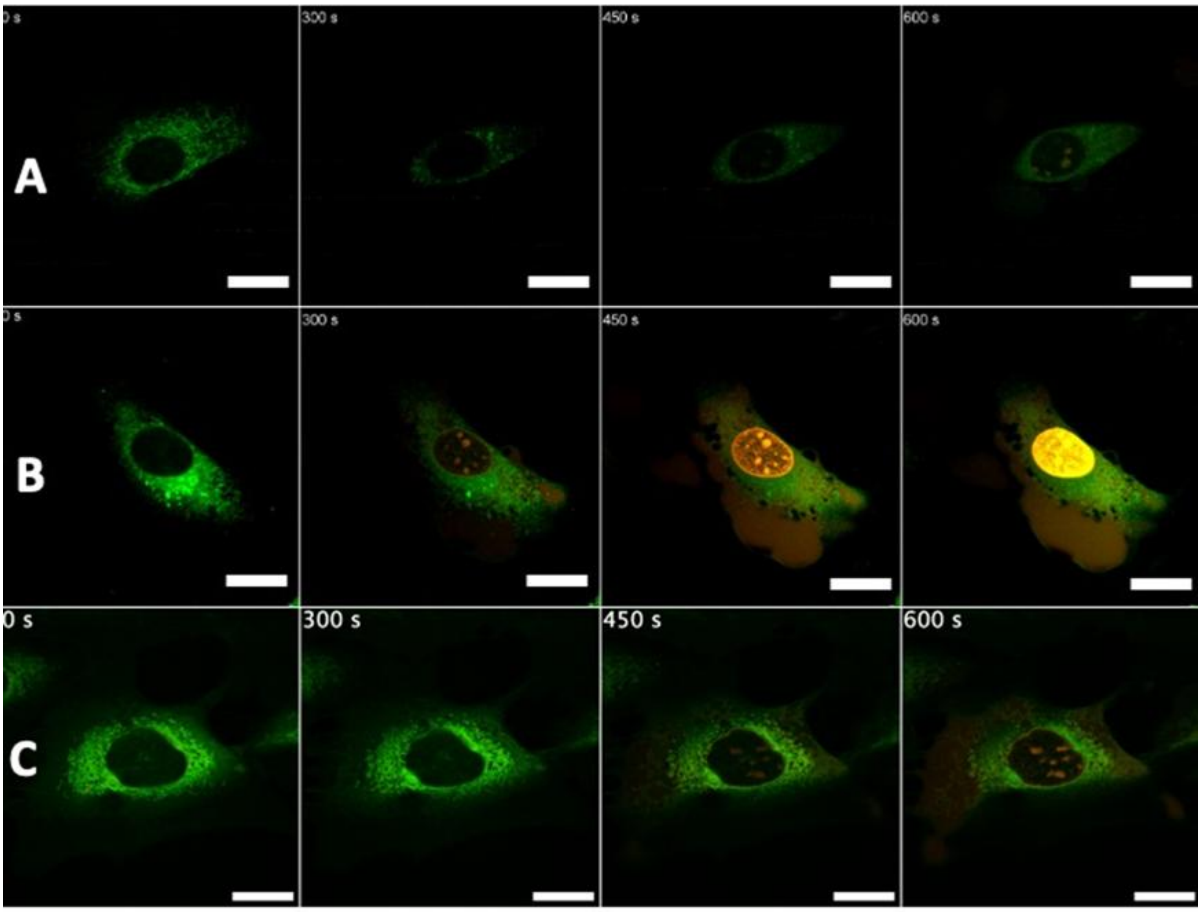

Figure S8 Microscopic observation of necrotic cell death caused by excitation at 355 nm (UV-exposure times are shown for each image), quantified by the observation of PI fluorescence within the nucleus. **A** – Control sample: NIH 3T3 cells loaded with 100 nM PI and 0.1% DMSO. **B** – NIH 3T3 cells loaded with 100 nM PI and 0.5 μM **MNM 1**. **C** - NIH 3T3 cells loaded with 100 nM PI and 0.5 μM **MNM 4**. All image sets collected after 30 minutes incubation after dosing procedure. Overlaid channels of PI fluorescence (λ_{ex} = 543 nm, 0.2 mW; λ_{em} = 600-700 nm), and mitochondrial autofluorescence (λ_{ex} = 355 nm, 20 mW, 400 nJ per voxel; λ_{em} = 440-460 nm). All scale bars refer to 20 μm.

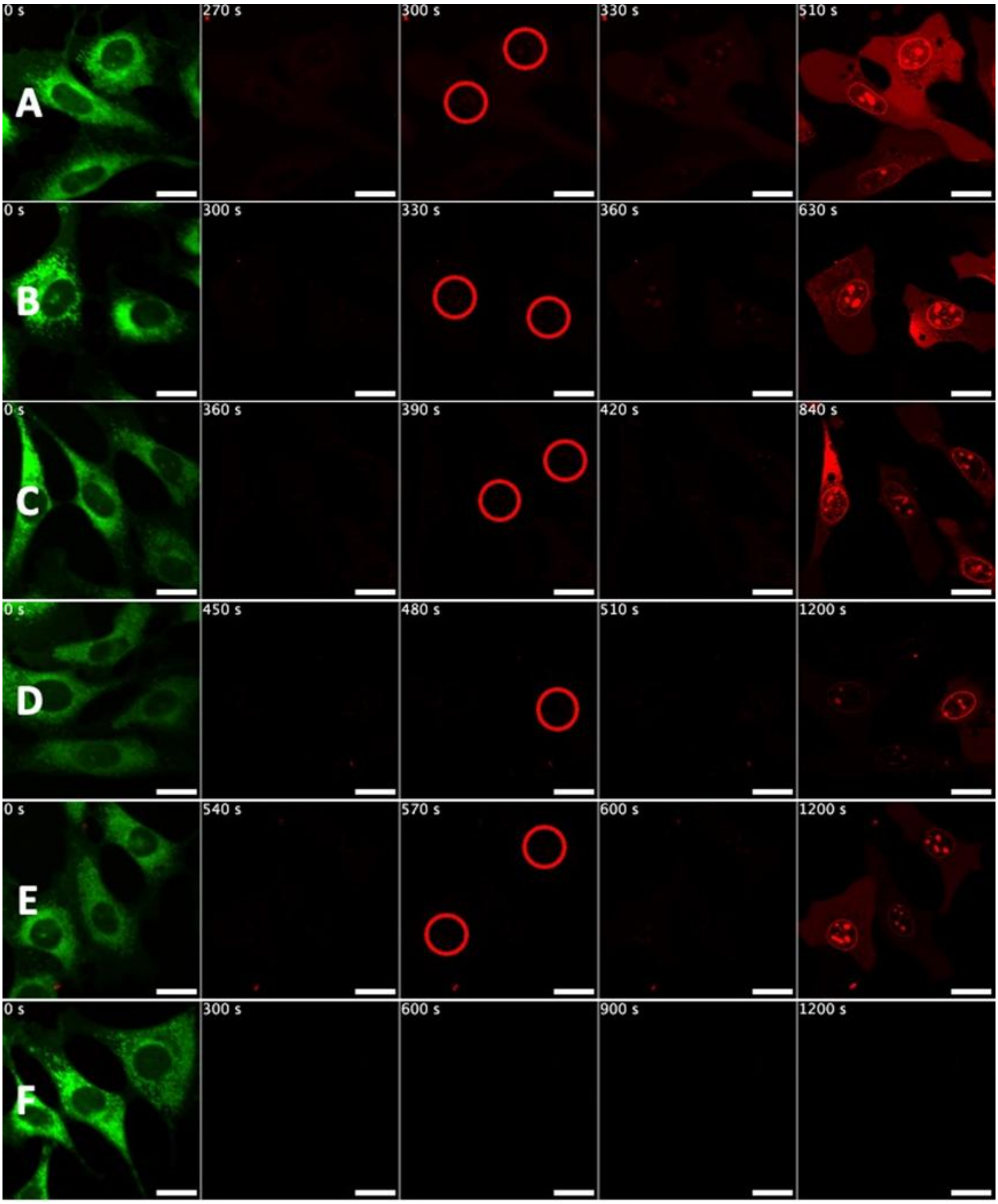

Figure S9 Microscopic observation of cell death caused by excitation at 355 nm (Experiment run times are shown for each image), quantified by the observation of PI fluorescence within the nucleus. **A** – NIH 3T3 cells loaded with 0.5 μM **MNM 4** followed by 2-hour incubation and washing with MNM free media, and subsequent staining with 100 nM PI. Exposed to 355 nm UV laser light for the entirety of the experiment. **B** – NIH 3T3 cells loaded with 0.5 μM **MNM 4** followed by 2-hour incubation and washing with MNM free media, and subsequent staining with 100 nM PI. 355 nm UV laser switched off after **5 minutes** of exposure. **C** - NIH 3T3 cells loaded with 0.5 μM **MNM 4** followed by 2-hour incubation and washing with MNM free media, and subsequent staining with 100 nM PI. 355 nm UV laser switched off after **4 minutes** of exposure. **D** - NIH 3T3 cells

loaded with 0.5 μM **MNM 4** followed by 2-hour incubation and washing with MNM free media, and subsequent staining with 100 nM PI. 355 nm UV laser switched off after **3 minutes** of exposure. **E** - NIH 3T3 cells loaded with 0.5 μM **MNM 4** followed by 2-hour incubation and washing with MNM free media, and subsequent staining with 100 nM PI. 355 nm UV laser switched off after **2 minutes** of exposure. **F** - NIH 3T3 cells loaded with 0.5 μM **MNM 4** followed by 2-hour incubation and washing with MNM free media, and subsequent staining with 100 nM PI. 355 nm UV laser switched off after **1 minutes** of exposure All image sets collected after 30 minutes incubation after staining procedure. Overlaid channels of PI fluorescence (λ_{ex} = 543 nm, 0.2 mW; λ_{em} = 600-700 nm), and mitochondrial autofluorescence (λ_{ex} = 355 nm, 20 mW, 400 nJ per voxel; λ_{em} = 440-460 nm). All scale bars refer to 20 μm. Red circles superimposed to illustrate the first observable emission of PI (600-700 nm) within the nucleus of the studied cells.

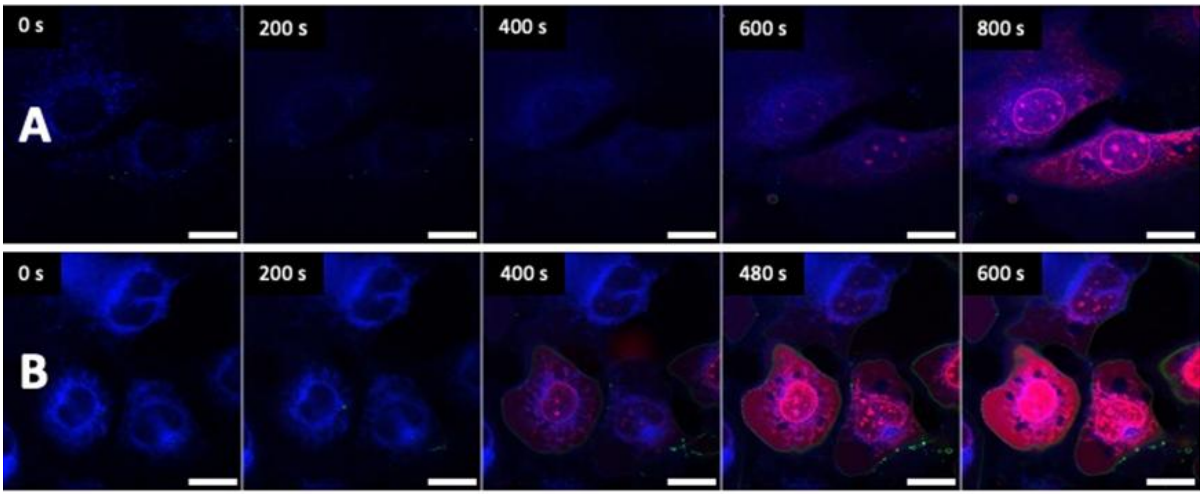

Figure S10 Microscopic observation of cell death caused by excitation at 355 nm (UV exposure times are shown for each image), quantified by the observation of PI fluorescence within the nucleus. **A** - Control sample: NIH 3T3 cells loaded with 100 nM PI, 100 nM AV, and 0.1% DMSO. **B** - NIH 3T3 cells loaded with 0.5 μM **MNM 1**, 100 nM PI, 100 nM AV, and 0.1% DMSO. All image sets collected after 30 minutes incubation after staining procedure. Overlaid channels of PI fluorescence (λ_{ex} = 543 nm, 0.2 mW; λ_{em} = 600-700 nm), AV fluorescence (λ_{ex} = 488 nm, 0.2 mW; λ_{em} = 500-550 nm) and mitochondrial autofluorescence (λ_{ex} = 355 nm, 20 mW, 400 nJ per voxel; λ_{em} = 440-460 nm). All scale bars refer to 20 μm.

Supplementary Synthetic Methods

MNM 1

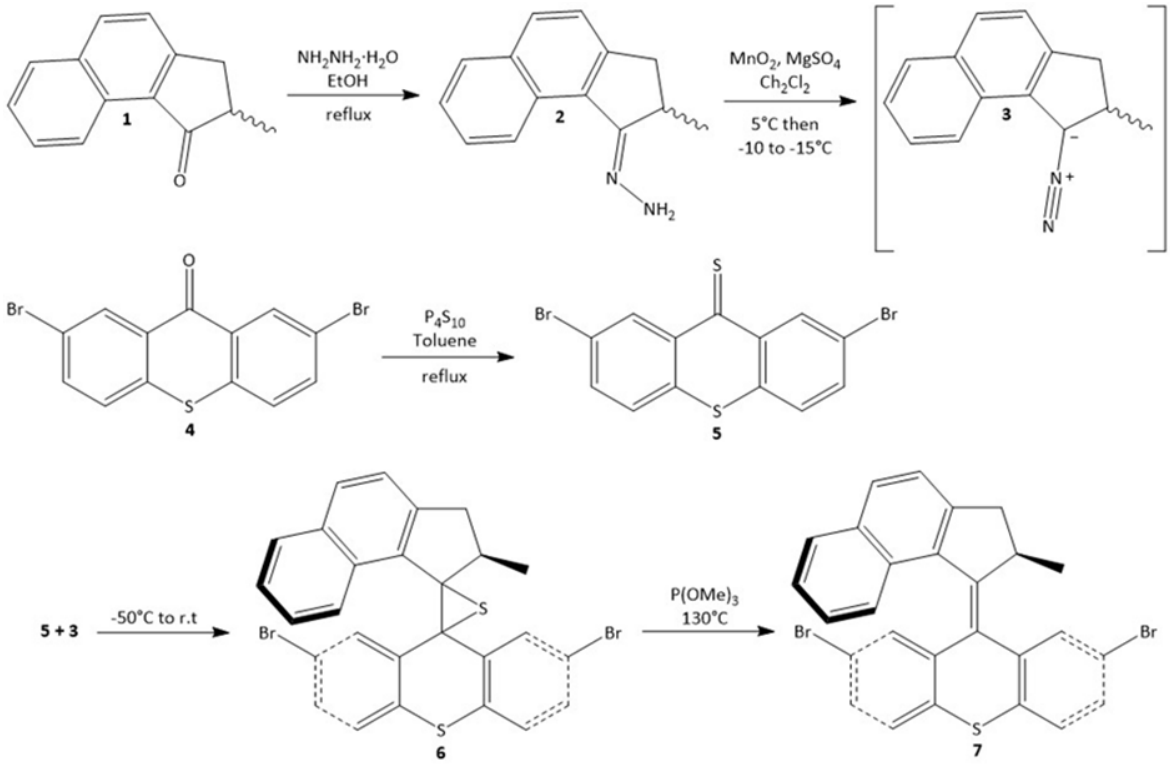

Scheme S1

Hydrazone 2. To an oven dried round-bottom flask charged with ketone 1 (1.96 g, 10 mmol) was added EtOH (20 mL) and hydrazine monohydrate (10 mL) through a condenser. The mixture was heated at reflux for 3 d. The cooled reaction mixture was extracted with ether (100 mL) and water (100 mL × 3). The organic layer was dried over MgSO4 and concentrated under vacuum to afford hydrazone 2 as yellow solid (1.95 g, 93%). Spectroscopic data were identical to those in the literature.[2]

2,7-Dibromo-thioxanthene-9-thione (5). To an oven dried two-neck round-bottom flask charged with thioxanthenone 4 12 (3.25 g, 8.78 mmol) and P_4S_{10} (10 g, 22.5 mmol) was added toluene (250 mL) and the mixture was heated at reflux for 2 d. The mixture was filtered while hot, and the filtrate was collected. The brownish solid that formed upon cooling was filtered, and solid was collected and dried under vacuum (3.2 g, 94 %). Spectroscopic data were identical to those reported in the literature.[2]

Episulfide 6. To an oven dried three-neck round-bottom flask charged with hydrazone 2 (0.99 g, 4.7 mmol) and $MgSO_4$(s) (0.49 g, 50% w/w) was added dichloromethane (25 mL). To this suspension was added quickly MnO_2 (1.62 g, 18.8 mmol, Sigma-Aldrich > 90%) at ca. 5 °C. The reaction flask was immediately immersed and stirred in a cold bath ranging from −15 °C to −10 °C for 1.5 h. After this period, the reaction mixture was cooled to −50 °C and then transferred to a Schlenk filtration tube connected to an oven dried three-neck round-bottom flask. The deep purple filtrate that contained intermediate 3 was collected, and the Schlenk tube was rinsed with pre-cooled

dichloromethane (20 mL, −50 °C). To the flask containing the combined filtrate, thione 5 (0.97 g, 2.5 mmol) was added portionwise until no more N_2 evolved. The mixture was stirred for an additional 0.5 h at ambient temperature. The mixture was poured into methanol (80 mL) with vigorous stirring and a white precipitate formed. The solid was filtered, and the filter cake was washed with methanol (30 mL) and dried under vacuum to afford the desired compound 6 (1.19 g, 84%): mp 204 °C (decomp); FTIR (neat) 3078, 3070, 3050, 2974, 2954, 2934, 2898, 2866, 2840, 1616, 1580, 1568, 1556, 1514, 1456, 1436, 1382, 1372, 1252, 1212, 1160, 1132, 1112, 1080, 1052, 1024 cm^{-1}. For NMR spectroscopic data, see reference 2.[2]

2,7-Dibromo-9-(2-methyl-2,3-dihydro-1H-cyclopenta[a]naphthalen-1-ylidene)-9H-thioxanthene (molecular motor 7). To a 200 mL screw-capped tube charged with episulfide 6 (524 mg, 0.96 mmol) was added trimethyl phosphite (9.6 mL), and the mixture was stirred at 130 °C for 14 h. After the reaction mixture was cooled to room temperature, methanol (30 mL) was added. The precipitate was filtered and washed with methanol (20 mL). The solid was purified by column chromatography on silica gel using hexanes:dichloromethane 9:1 as eluent to afford compound 7 as a pale yellow solid (485 mg, 94%): mp 245–246 °C; FTIR (neat) 3072, 3046, 3032, 3008, 2952, 2920, 2859, 2848, 1616, 1612, 1576, 1568, 1558, 1548, 1540, 1512, 1452, S5 1440, 1394, 1380, 1362, 1346, 1284, 1256, 1204, 1196, 1180, 1152, 1140, 1128, 1084, 1074, 1052 cm^{-1} ; HRMS (APCI) m/z calculated for for [M+H]+ C27H19Br2S 532.9574, found 532.9550. For NMR spectroscopic data, see reference 2.[2]

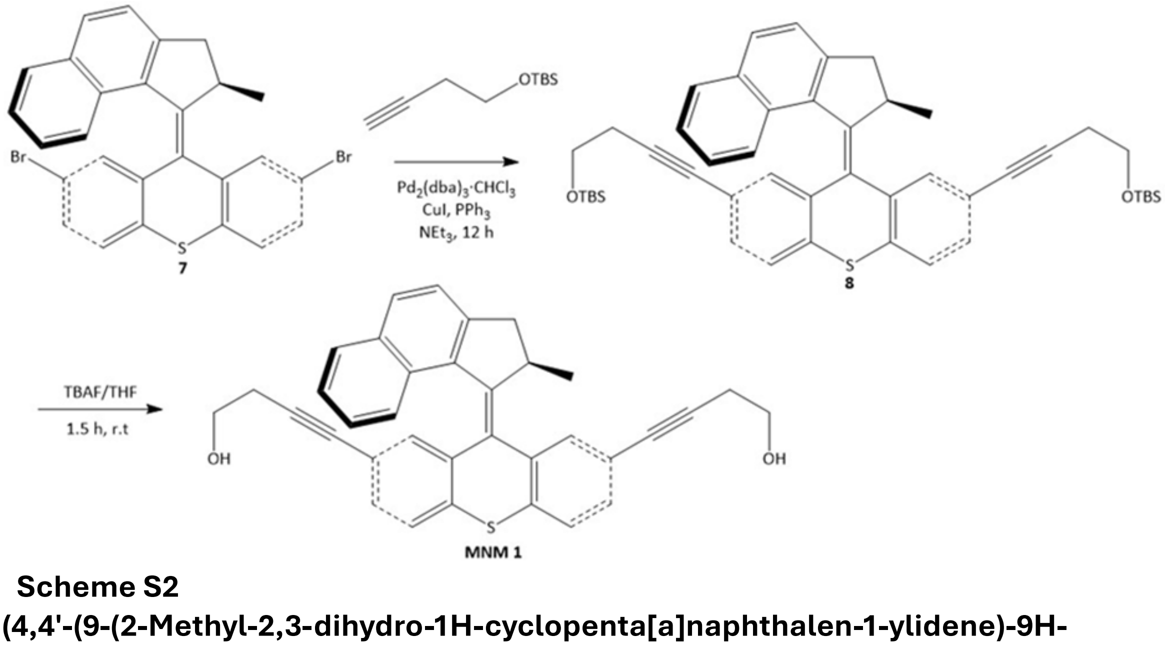

Scheme S2

(4,4'-(9-(2-Methyl-2,3-dihydro-1H-cyclopenta[a]naphthalen-1-ylidene)-9H-thioxanthene-2,7-diyl)bis(but-3-yne-4,1-diyl))bis(oxy)bis(tert-butyldimethylsilane) (molecular motor 8). An oven dried Schlenk tube equipped with a stir bar was charged with motor 7 (750 mg, 1.4 mmol), tris(dibenzylideneacetone)dipalladium(0)-chloroform adduct (25.6 mg, 0.028 mmol), CuI (5.4 mg, 0.028 mmol), triphenylphosphine (26.3 mg, 0.14 mmol) and 4-(tert-butyldimethylsilyloxy)- but-1-yne (1.44 ml, 7.0 mmol). NEt3 (7 mL) was added and the mixture was stirred at 70 °C overnight. The resulting mixture was partitioned between CH_2Cl_2 (40 mL) and saturated NH_4Cl (aq) (40 mL). The organic layer was dried over

anhydrous $MgSO_4$, concentrated, and purified by column chromatography (silica gel; 30% CH2Cl2 in hexanes) to afford 8 as a pale yellow solid (915 mg, 90%): m.p. 207–209 °C; FTIR (neat) 3052, 2954, 2930, 2856, 1588, 1472, 1454, 1388, 1362, 1252, 1218, 1100, 1058, 1006 cm^{-1}. For NMR spectroscopic data, see reference 3.[3]

4,4'-(9-(2-Methyl-2,3-dihydro-1H-cyclopenta[a]naphthalen-1-ylidene)-9H-thioxanthene-2,7-diyl)dibut-3-yn-1-ol (MNM 1). A 100 mL round-bottomed flask equipped with a stir bar was charged with molecular motor 8 (544 mg, 0.73 mmol). THF (10 mL) and a solution of TBAF (1.83 mL, 1.83 mmol, 1.0 M in THF) were added, and the mixture was stirred at rt for 1.5 h. The mixture was poured into water (50 mL) and filtered. The solid was collected, washed with water (20 mL ×2) and dried under vacuum to afford desired product MNM 1 as a pale-yellow solid (358 mg, 95%): m.p. 240 °C (decomposition.); FTIR (neat) 3302, 3050, 2954, 2922, 2894, 2838, 1702, 1586, 1516, 1454, 1386, 1338, 1256, 1170, 1042, 1020 cm^{-1}. For NMR spectroscopic data, see reference 3.

MNM 2

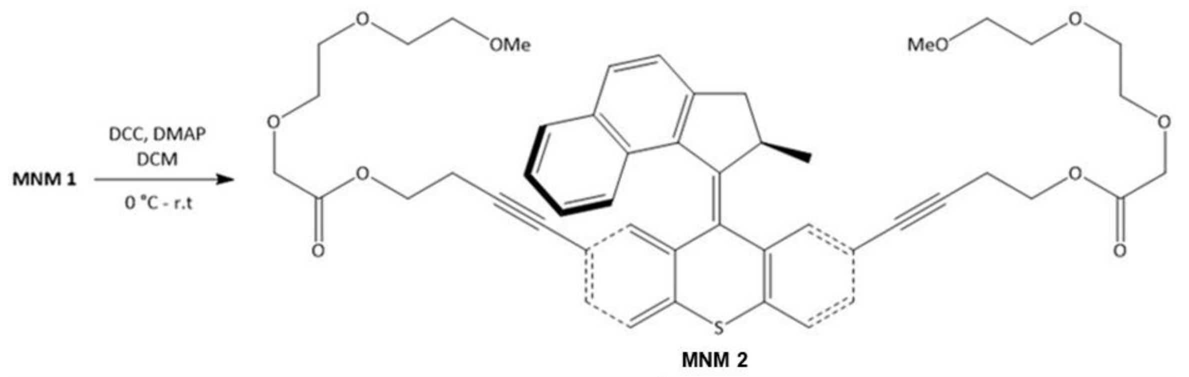

Scheme S3

(9-(2-methyl-2,3-dihydro-1H-cyclopenta[a]naphthalen-1-ylidene)-9Hthioxanthene-2,7-diyl)bis(but-3-yne-4,1-diyl)bis(2-(2-(2-methoxyethoxy)ethoxy)acetate) (MNM 2). An oven dried round-bottom flask equipped with a stir bar was charged with MNM 1 (12 mg, 0.022 S29 mmol), 2-(2-(2-methoxyethoxy)ethoxy)acetic acid (0.02 mL, excess), DCC (4.5 mg, 0.022 mml), DMAP (0.3 mg, 0.0022 mmol) and DCM (5 mL) at 0 °C. The suspension was stirred vigorously for 18 h at rt. The resulting yellow solution was partitioned between DCM (20 mL) and water (20 mL). The organic phase was dried over anhydrous $MgSO_4$, filtered and the filtrate was concentrated in vacuo, followed by purification by column chromatography (SiO_2; 20% acetone in DCM) to afford MNM 2 as a bright yellow solid (15.9 mg, 84%). For NMR spectroscopic data, see reference 4.[4]

MNM 3

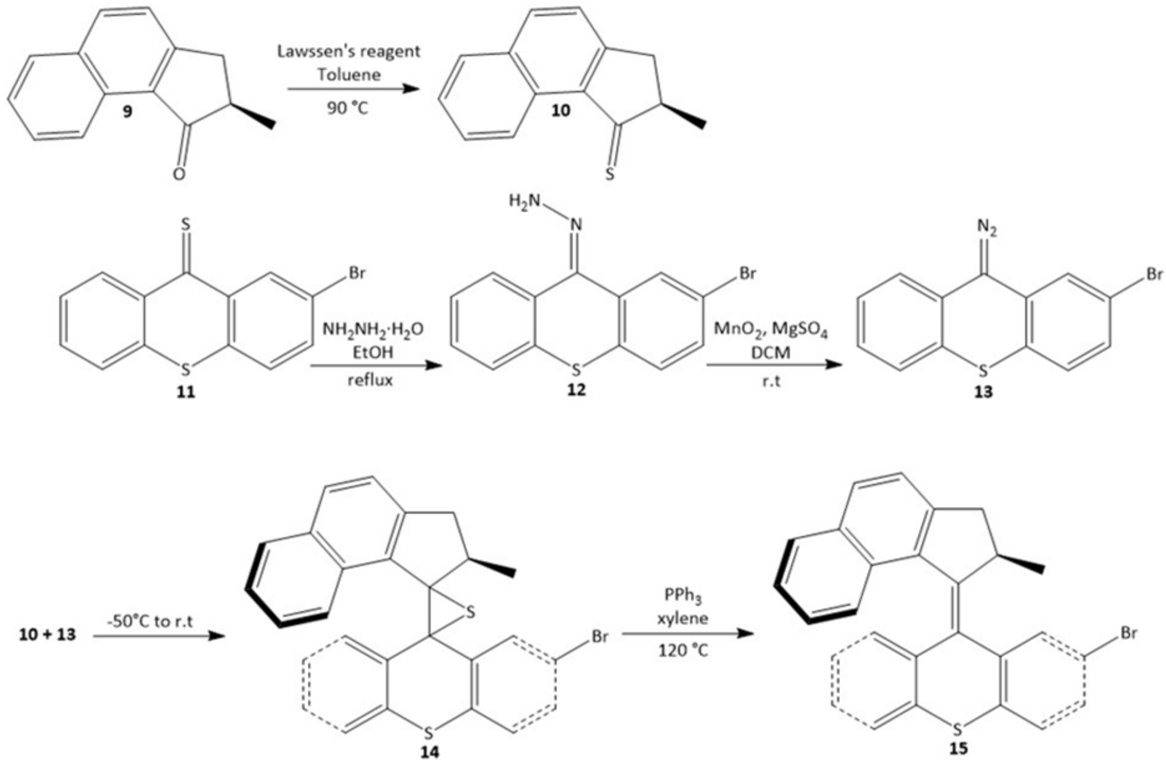

Scheme S4

(9H-thioxanthen-9-ylidene)hydrazine (12). An oven-dried round-bottom flask equipped with a stir bar was charged with compound 11 (50 mg, 0.221 mmol) in THF (5.0 ml), hydrazine monohydrate (1.0 ml) was added, and the mixture was stirred at room temperature for 1 h. The resulting mixture was concentrated in vacuo. The resulting concentrate was used for the next step without further purification.[5]

5-bromo-2-methyl-2,3-dihydrodispiro[cyclopenta[a]naphthalene-1,2'-thiirane-3',9''-thioxanthene] (14). To an oven-dried round-bottom flask charged with hydrazone 12 and $MgSO_4$ (100 mg, 200% w/w) was added THF (5.0 mL). To this suspension was quickly added MnO_2 (500.0 mg, 5.7 mmol, Sigma-Aldrich > 90%) at room temperature. The mixture was stirred for 1 h at the same temperature. The mixture was filtered, and the filtrate was concentrated in vacuo. To the resulting concentrate was added toluene (5.0 mL) and thioketone 10 (34.6 mg, 0.119 mmol). The mixture was heated to 100 °C and stirred for 3 h. After the reaction mixture was cooled to room temperature, the organic phase was dried over anhydrous MgSO4, filtered, and the filtrate was concentrated in vacuo, followed by purification by column chromatography (SiO_2; 10% acetone in DCM) to yield 20 as a bright yellow solid (38.6 mg, 67% for 2 steps). For NMR spectroscopic data, see reference 6.[6]

9-(5-bromo-2-methyl-2,3-dihydro-1H-cyclopenta[a]naphthalen-1-ylidene)-9H-thioxanthene (15). To a 100 mL screw-capped tube charged with episulfide 14 (101 mg, 0.208 mmol) was added triphenylphosphine (108 mg, 0.416 mmol), and the mixture was stirred at 140 °C for 14 h. After the reaction mixture was cooled to room temperature, the resulting mixture was partitioned between DCM (10 mL) and saturated NH_4Cl (aq) (10 mL). The organic layer was dried over anhydrous $MgSO_4$, filtered, and the filtrate concentrated in vacuo. The resulting concentrate was purified

by column chromatography (silica gel; 20% DCM in hexanes) to yield 15 as a pale yellow solid (86.4 mg, 89%). For NMR spectroscopic data, see reference 6.[6]

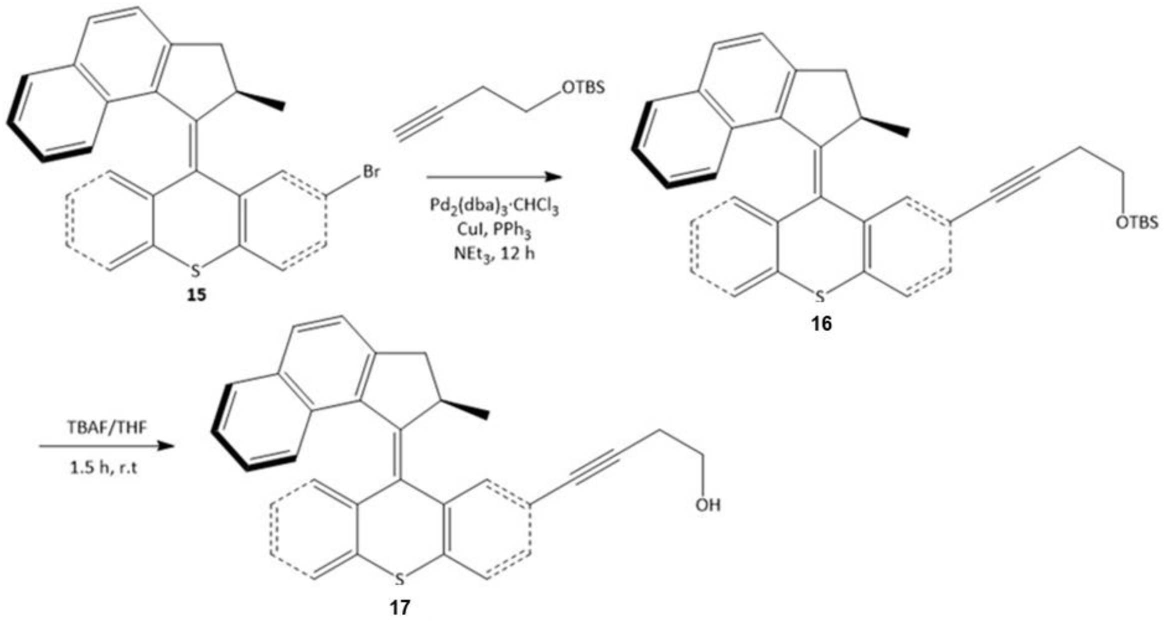

Scheme S5

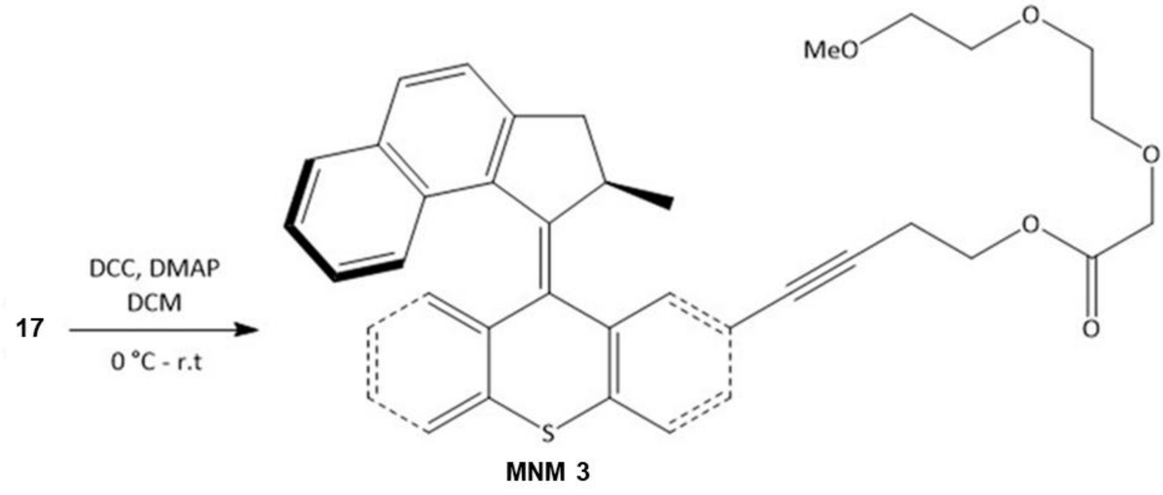

Scheme S6

4-(9-(2-methyl-2,3-dihydro-1H-cyclopenta[a]naphthalen-1-ylidene)-9H-thioxanthen-2-yl)but-3-yn-1-yl2-(2-(2-methoxyethoxy)ethoxy)acetate (MNM 3). An oven dried round-bottom flask equipped with a stir bar was charged with 17 (16 mg, 0.029 mmol), 2-(2-(2- methoxyethoxy)ethoxy)acetic acid (0.03 ml, excess), DCC (5.5 mg, 0.029 mml), DMAP (0.5 mg, 0.0029 mmol) and DCM (5 mL) at 0 °C. The suspension was stirred vigorously for 18 h at rt. The resulting yellow solution was partitioned between DCM (20 mL) and water (20 mL). The organic phase was dried over anhydrous $MgSO_4$, filtered and the filtrate was concentrated in vacuo, followed by purification by column chromatography (SiO2; 20% acetone in DCM) to afford MNM 3 as a bright yellow solid (16.7 mg, 81%). For NMR spectroscopic data, see reference 7.[7]

MNM 4

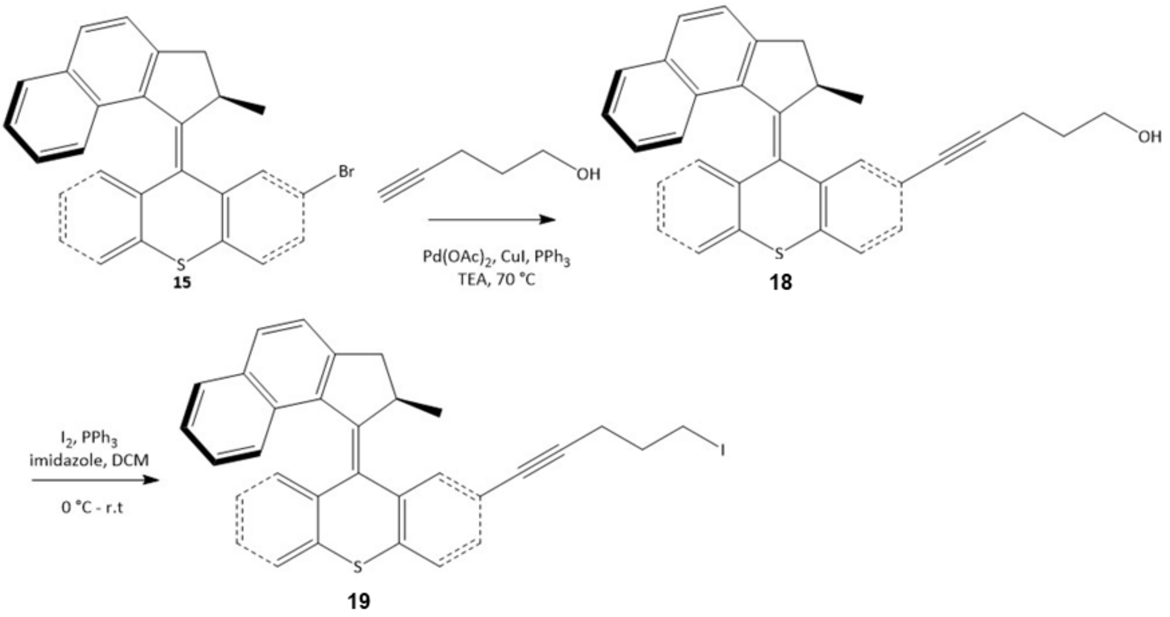

Scheme S7

5-(9-(2-methyl-2,3-dihydro-1H-cyclopenta[a]naphthalen-1-ylidene)-9Hthioxanthen-2-yl)pent-4-yn-1-ol (18). An oven dried round-bottom flask equipped with a stir bar was charged with motor 15 (50 mg, 0.11 mmol), palladium(II) acetate (2.5 mg, 0.011 mmol), CuI (2.0 mg, 0.011 mmol), PPh_3 (5.8 mg, 0.022 mmol) and but-4-yn-1- ol (0.03 mL, 0.44 mmol). NEt_3 (3 mL) was added and the mixture was stirred at 70 °C overnight. The resulting mixture was partitioned between DCM (10 mL) and saturated NH_4Cl (aq) (10 mL). The organic layer was dried over anhydrous $MgSO_4$, filtered and the filtrate concentrated in vacuo. The resulting concentrate was purified by column chromatography (silica gel; 30% DCM in hexanes) to afford 18 as a pale yellow solid (39.5 mg, 78%). For NMR spectroscopic data, see reference 8.[8]

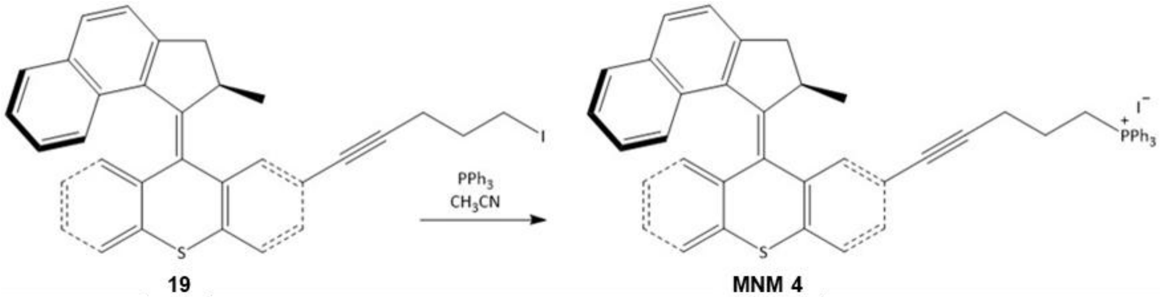

Scheme S8

(5-(9-(2-methyl-2,3-dihydro-1H-cyclopenta[a]naphthalen-1-ylidene)-9Hthioxanthen-2-yl)pent-4-yn-1-yl)triphenylphosphonium iodide (MNM 4). An oven dried round-bottom flask equipped with a stir bar was charged with motor 18 (25 mg, 0.055 mmol), iodine (28 mg, 0.11 mmol) and imidazole (7.5 mg, 0.11 mmol) at 0°C. PPh_3 (28.8 mg, 0.11 mmol) was added slowly at 0°C and the mixture was stirred at room temperature for 30min. Saturated solutions of sodium thiosulfate (10 mL) and sodium bicarbonate (10 mL) were added to the reaction mixture. The organic phase was separated, and the aqueous phase was extracted with methylene chloride. The

combined organic layers were washed with brine, dried over $MgSO_4$, and then concentrated in vacuo. The resulting concentrate was purified by flash column chromatography and the resulting iodide product 19 was used for the next step due to its instability. To a stirred solution of compound 27 in dry acetonitrile (4 mL) at room temperature, PPh_3 (56 mg, 0.22 mmol) was added, and then the mixture was allowed to stir under reflux for 48 h. After TLC analysis indicated the consumption of the starting material, the solvent was subsequently removed under reduced pressure, and the residue was purified by flash chromatography to afford MNM 4 as a white solid (19.5 mg, 43% for two steps). For NMR spectroscopic data, see reference 8.[8]

References

(1) Liu, D.; García-López, V.; Gunasekera, R. S.; Greer Nilewski, L.; Alemany, L. B.; Aliyan, A.; Jin, T.; Wang, G.; Tour, J. M.; Pal, R. Near-Infrared Light Activates Molecular Nanomachines to Drill into and Kill Cells. *ACS Nano* **2019**, *13* (6), 6813–6823. https://doi.org/10.1021/acsnano.9b01556.

(2) Chiang, P.-T.; Mielke, J.; Godoy, J.; Guerrero, J. M.; Alemany, L. B.; Villagómez, C. J.; Saywell, A.; Grill, L.; Tour, J. M. Toward a Light-Driven Motorized Nanocar: Synthesis and Initial Imaging of Single Molecules. *ACS Nano* **2012**, *6* (1), 592–597. https://doi.org/10.1021/nn203969b.

(3) García-López, V.; Chiang, P.-T.; Chen, F.; Ruan, G.; Martí, A. A.; Kolomeisky, A. B.; Wang, G.; Tour, J. M. Unimolecular Submersible Nanomachines. Synthesis, Actuation, and Monitoring. *Nano Lett.* **2015**, *15* (12), 8229–8239. https://doi.org/10.1021/acs.nanolett.5b03764.

(4) Ayala Orozco, C.; Liu, D.; Li, Y.; Alemany, L. B.; Pal, R.; Krishnan, S.; Tour, J. M. Visible-Light-Activated Molecular Nanomachines Kill Pancreatic Cancer Cells. *ACS Appl. Mater. Interfaces* **2020**, *12* (1), 410–417. https://doi.org/10.1021/acsami.9b21497.

(5) Santos, A. L.; Liu, D.; Reed, A. K.; Wyderka, A. M.; van Venrooy, A.; Li, J. T.; Li, V. D.; Misiura, M.; Samoylova, O.; Beckham, J. L.; Ayala-Orozco, C.; Kolomeisky, A. B.; Alemany, L. B.; Oliver, A.; Tegos, G. P.; Tour, J. M. Light-Activated Molecular Machines Are Fast-Acting Broad-Spectrum Antibacterials That Target the Membrane. *Science Advances* **2022**, *8* (22), eabm2055. https://doi.org/10.1126/sciadv.abm2055.

(6) Santos, A. L.; Liu, D.; Reed, A. K.; Wyderka, A. M.; van Venrooy, A.; Li, J. T.; Li, V. D.; Misiura, M.; Samoylova, O.; Beckham, J. L.; Ayala-Orozco, C.; Kolomeisky, A. B.; Alemany, L. B.; Oliver, A.; Tegos, G. P.; Tour, J. M. Light-Activated Molecular Machines Are Fast-Acting Broad-Spectrum Antibacterials That Target the Membrane. *Science Advances* **2022**, *8* (22), eabm2055. https://doi.org/10.1126/sciadv.abm2055.

(7) Ayala Orozco, C.; Liu, D.; Li, Y.; Alemany, L. B.; Pal, R.; Krishnan, S.; Tour, J. M. Visible-Light-Activated Molecular Nanomachines Kill Pancreatic Cancer Cells. *ACS Appl. Mater. Interfaces* **2020**, *12* (1), 410–417. https://doi.org/10.1021/acsami.9b21497.

(8) Galbadage, T.; Liu, D.; Alemany, L. B.; Pal, R.; Tour, J. M.; Gunasekera, R. S.; Cirillo, J. D. Molecular Nanomachines Disrupt Bacterial Cell Wall, Increasing Sensitivity of Extensively Drug-Resistant Klebsiella Pneumoniae to Meropenem. *ACS Nano* **2019**, *13* (12), 14377–14387. https://doi.org/10.1021/acsnano.9b07836.
